# Development and evaluation of rapid and accurate one-tube RPA-CRISPR-Cas12b-based detection of *mcr-1* and *tet*(X4)

**DOI:** 10.1007/s00253-024-13191-6

**Published:** 2024-05-27

**Authors:** Yu Wang, Huan Chen, Qingyun Pan, Jing Wang, Xin’an Jiao, Yunzeng Zhang

**Affiliations:** 1 Jiangsu Co‐Innovation Center for Prevention and Control of Important Animal Infectious Diseases and Zoonoses, Yangzhou University, Yangzhou, 225009 China; 2 Jiangsu Key Laboratory of Zoonosis, Yangzhou University, Yangzhou, 225009 China; 3 Joint International Research Laboratory of Agriculture and Agri‐product Safety of the Ministry of Education, Yangzhou University, Yangzhou, 225009 China; 4 Key Laboratory of Prevention and Control of Biological Hazard Factors (Animal Origin) for Agrifood Safety and Quality, Ministry of Agriculture of China, Yangzhou University, Yangzhou, 225009 China

**Keywords:** RPA; CRISPR, Cas12b; *Mcr*, *1*; *Tet*(X4); Detection

## Abstract

**Abstract:**

The emergence and quick spread of the plasmid-mediated tigecycline resistance gene *tet*(X4) and colistin resistance gene *mcr-1* have posed a great threat to public health and raised global concerns. It is imperative to develop rapid and accurate detection systems for the onsite surveillance of *mcr-1* and *tet*(X4). In this study, we developed one-tube recombinase polymerase amplification (RPA) and CRISPR-Cas12b integrated *mcr-1* and *tet*(X4) detection systems. We identified *mcr-1-* and *tet*(X4)-conserved and -specific protospacers through a comprehensive BLAST search based on the NCBI nt database and used them for assembling the detection systems. Our developed one-tube RPA-CRISPR-Cas12b-based detection systems enabled the specific detection of *mcr-1* and *tet*(X4) with a sensitivity of 6.25 and 9 copies within a detection time of ~ 55 and ~ 40 min, respectively. The detection results using pork and associated environmental samples collected from retail markets demonstrated that our developed *mcr-1* and *tet*(X4) detection systems could successfully monitor *mcr-1* and *tet*(X4), respectively. Notably, *mcr-1-* and *tet*(X4)-positive strains were isolated from the positive samples, as revealed using the developed detection systems. Whole-genome sequencing of representative strains identified an *mcr-1*-carrying IncI2 plasmid and a *tet*(X4)-carrying IncFII plasmid, which are known as important vectors for *mcr-1* and *tet*(X4) transmission, respectively. Taken together, our developed one-tube RPA-CRISPR-Cas12b-based *mcr-1* and *tet*(X4) detection systems show promising potential for the onsite detection of *mcr-1* and *tet*(X4).

**Key points:**

*• One-tube RPA-CRISPR-Cas12b-based mcr-1 and tet(X4) detection systems were developed based on identified novel protospacers.*

*• Both detection systems exhibited high sensitivity and specification with a sample-to-answer time of less than 1 h.*

*• The detection systems show promising potential for onsite detection of mcr-1 and tet(X4).*

**Supplementary Information:**

The online version contains supplementary material available at 10.1007/s00253-024-13191-6.

## Introduction

Antibiotics have been widely used in animal husbandry for recent several decades as an important means to treat and prevent microbial infections and promote animal growth (Woolhouse and Ward 2013; Zhu et al. 2017). However, the long-term overuse of antibiotics has been causing the emergence and spread of antibiotic-resistant pathogens, particularly multidrug-resistant ones, in livestock and poultry farms and their meat products (Serwecińska 2020; Teillant and Laxminarayan 2015; VT Nair et al. 2018). Such contaminated meat products could lead to infections in humans and cause diseases, such as diarrhea, which can seriously threaten public health (Exner et al. 2017; Manyi-Loh et al. 2018). The increasing spread of antibiotic-resistant pathogens has posed great challenges to the treatment of microbial diseases using antibiotics.

In the absence of new suitable antibiotics, colistin and tigecycline are currently the “last line of defense” antibiotics for the clinical treatment of multidrug-resistant Gram-negative bacteria (Lu et al. 2021; Osei et al. 2016). However, in recent years, their antibiotic-resistance genes (ARGs) have also been continuously discovered and reported. In 2015, Liu et al. first discovered a plasmid-mediated colistin resistance gene from animal-derived *Escherichia coli* and named it *mcr-1* (Liu et al. 2016). They simultaneously proved that *mcr-1* could be transferred to *Klebsiella pneumoniae* through the IncI2 conjugative plasmid and mediate colistin resistance in the receipt strain. Since then, at least 10 homologues of *mcr* have been described; however, *mcr-1* is the most prevalent *mcr* variant identified in clinics worldwide (Dadashi et al. 2022). Among the identified *tet*(X) variants that can degrade tigecycline, *tet*(X4) is recognized as a highly prevalent plasmid-borne variant that exhibits high enzymatic activity and can mediate high resistance to tigecycline (16–32 mg/mL) (He et al. 2019). To date, *tet*(X4)-positive strains have been identified in animals (Wang et al. 2022a, b), foods (Sun et al. 2021), the environment (Cui et al. 2020), and humans (Cui et al. 2022; Zhang et al. 2022a, b). Gradually, these strains are increasingly being reported worldwide.

The rapid spread of *mcr-1* and *tet*(X4), which is exacerbated by the self-replication and transfer of bacterial interstitial plasmids, has raised serious concerns regarding the safety of livestock and poultry industries and human health (He et al. 2019; Liu et al. 2016). The food chain, one of the main transmission routes of ARGs, is responsible for the transmission of *mcr-1* and *tet*(X4) to humans through livestock and poultry meat products. This may have adverse effects on human health, such as the imbalance of gut microbiota and the proliferation of antibiotic-resistant pathogens (de Mesquita et al. 2022). A study on the prevalence of *mcr-1* in *Enterobacteriaceae* species isolated from the United Kingdom (UK) identified four *mcr-1*-positive human-derived *Salmonella* strains that shared the same ST type as swine-derived *Salmonella* strains reported in a UK farm, indicating the potential transmission of these four strains through the food chain, particularly through animal food (Doumith et al. 2016). Similarly, *tet*(X4) has been isolated from various animals, such as pigs, chickens, and ducks, in more than 23 countries, with pigs being the primary source of animal isolation (Zhang et al. 2022a, b). For instance, Sun et al. identified 25 *tet*(X4)-positive strains from a total of 311 retail meat samples, with pork accounting for 52% of the isolates (Sun et al. 2021). Moreover, in a study conducted in Singapore, 10.1% of 109 residents carried *tet*(X4)-positive strains in their feces, indicating that *tet*(X4) has been widely distributed in the gut microbiota of healthy residents in Singapore (Zhang et al. 2022a, b). Alarmingly, an increasing number of studies have revealed the coexistence of *tet*(X4) and *mcr-1* in *E. coli* (Li et al. 2022a, b, c, d, e). The convergence of resistance to “last line of defense” antibiotics can lead to the potential emergence of superbugs in the future. Therefore, the rapid and accurate onsite detection of *mcr-1* and *tet*(X4) in food chain-associated samples, such as meat and associated environments, is crucial for ensuring food safety and maintaining human health.

In recent years, various methods have been developed for the detection of *mcr-1* and *tet*(X4); these include cultivation-based assays, PCR-based detection techniques (e.g., PCR-dipstick chromatography and real-time quantitative PCR assays) (Coppi et al. 2018; Shanmugakani et al. 2019; Chabou et al. 2016; Li et al. 2020), and other modern molecular diagnostic techniques. Nonetheless, despite its advantages in visualization and reliability, the cultivation-based assays are characterized by low sensitivity and time-consuming procedures. Concurrently, PCR-based techniques, while possessing high specificity, may presently be unsuitable for onsite detection due to their reliance on specialized amplification instruments (e.g., a thermocycler) and trained laboratory professionals. Recently, the CRISPR-Cas-based nucleic acid detection technology has shown promising potential for rapid onsite pathogen and ARG detection. This technology offers several advantages, including ease of operation, independence from specialized amplification instruments, and superior accuracy and specificity compared to other molecular detection technologies (Li et al. 2023; Zhao et al. 2023; Wang et al. 2022a, b). Cas proteins, such as Cas12a and Cas12b, exhibit collateral cleavage activity that can activate the cleavage domain upon target nucleic acid cleavage, leading to the unrestricted cleavage of free nucleic acids present in the system (Li et al. 2019). Based on this principle, the results can be determined by the presence or absence of fluorescence by adding free fluorescent probes to the system. Cas12b, having a molecular weight inferior to that of Cas9 and Cas12a, is one of the most widely used Cas proteins for developing CRISPR-Cas-based nucleic acid detection systems. Cas12b exhibits a preference for recognizing protospacer sequences with the PAM site “TTA” (Yang et al. 2016). Moreover, its diminutive size coupled with a low tolerance for even a single-base mismatch renders it a favorable candidate for nucleic acid detection in vitro (Varshney et al. 2021). Currently, Cas12b has been used to rapidly and accurately detect pathogenic bacteria, such as *Campylobacter jejuni* (sample-to-answer time < 40 min) (Huang et al. 2021), *Mycobacterium tuberculosis* complex (< 70 min) (Yang et al. 2023), *Pseudomonas aeruginosa* (< 1 h) (Qiu et al. 2023), and *Klebsiella pneumoniae* (Qiu et al. 2022). Moreover, nucleic acid amplification technologies, such as recombinase polymerase amplification (RPA), which is a rapid amplification technology that can exponentially amplify the target sequence within 30 min, can be applied to the CRISPR-Cas-based detection systems to further enhance the specificity and sensitivity of detection (Hijjawi et al. 2023). The combination of RPA and CRISPR-Cas12a can enable the rapid detection of methicillin-resistant *Staphylococcus aureus*, achieving the detection of as low as 10 copies of the target gene within only 20 min (Li et al. 2022a, b, c, d, e). In this study, we developed an RPA-CRISPR-Cas12b-based detection technique to offer a novel approach for the rapid and accurate onsite identification of ARGs, such as *mcr-1* and *tet*(X4).

The combination of a CRISPR-Cas-based detection system and RPA is typically achieved by adding reagents in a stepwise manner and waiting for RPA to react before introducing CRISPR components (Lin et al. 2022). However, this approach may result in aerosol contamination and generate false-positive results (Lin et al. 2022). Moreover, the stepwise approach lacks convenience for onsite detection purposes. In contrast, colocalizing RPA- and CRISPR-Cas-based detection assays within the same reaction vessel can mitigate the risk of cross-contamination resulting from the opening and closing of PCR tubes during the experimental process (Hu et al. 2022). In this study, specific and conserved protospacer sequences used for the CRISPR-Cas12b assay were identified in *mcr-1* and *tet*(X4) by a comprehensive BLAST search based on the NCBI nt database, and the primer pairs for the RPA assay were then designed. Finally, one-tube RPA- and CRISPR-Cas12b-colocalized *mcr-1* and *tet*(X4) detection systems were developed. The feasibility of the two developed systems was assessed based on extracted DNA samples, artificially contaminated pork samples, and pork and their associated environmental samples collected from retail markets. The one-tube RPA-CRISPR-Cas-based *mcr-1* and *tet*(X4) detection systems accepted crude DNA prepared by boiling, thereby eliminating the need for bacterial isolation and DNA purification. The turnaround time from sample to result was ~ 55 min and ~ 40 min for *mcr-1* and *tet*(X4), respectively. The developed one-tube RPA-CRISPR-Cas-based detection systems showed promising potential for the onsite detection of *mcr-1* and *tet*(X4).

## Materials and methods

### Strains and oligonucleotides

The *tet*(X4)-positive strain *E. coli* 3934, the *mcr-1*-positive strain *E. coli* MCE9, and several other *tet*(X4)- and *mcr-1*-negative strains, including *E. coli* ATCC25922, *C. jejuni* NCTC11168, *S. aureus* ATCC27217, *Salmonella* Enteritidis C50041, *S.* Typhimurium SL1344, and *Listeria monocytogenes* EGD-e, were used for assessing the CRISPR-Cas12b-based *mcr-1* and *tet*(X4) detection systems.

DNA oligonucleotides and ssDNA-FQ fluorescent probes were synthesized by GenScript Biotechnology (Nanjing, China) (Table 1).

**Table 1:** Oligonucleotides used in this study

Oligo names	Sequence (5′-3′)
mcr-RPA-1F	CGACCAACAACGCCATCTGCAACACCAATCCTT
mcr-RPA-1R	TCATCATATCGCTTAAAATACGCAGGCCCGTGA
mcr-RPA-2F	ACGAATGCCGCGATGTCGGTATGCTCGTTGGC
mcr-RPA-2R	GATGTTCGCACTTGGCAAGCTCATTACCTTCA
mcr-RPA-3F	AACAACGCCATCTGCAACACCAATCCTTATA
mcr-RPA-3R	ATGTTCGCACTTGGCAAGCTCATTACCTTCA
tet-RPA-1F	TTGGGACGAACGCTACAAAGAACTGATTCGT
tet-RPA-1R	CATCAACCCGCTGTTTACGCCTTGTCCTGCA
tet-RPA-2F	GATTGGGACGAACGCTACAAAGAACTGATTC
tet-RPA-2R	GCATCCATCAACCCGCTGTTTACGCCTTGT
tet-RPA-3F	AACTGATTCGTGTGACATCATCTTTTGTAGG
tet-RPA-3R	GCTGTTAAATTTCCCATTGGTCAGATTATC
Protospacer-*mcr-1*	GATGACTTTGTCGCTGCCAA
Protospacer-*tet*(X4)	GGTAAGTCTTGGAAAAGTAA
mcr-sg-F	GAAATTAATACGACTCACTATAGGG
mcr-sg-R	TTGGCAGCGACAAAGTCATCGTGCCACTTCTCAGATTTGAGAAG
tet-sg-F	GAAATTAATACGACTCACTATAGGG
tet-sg-R	TTACTTTTCCAAGACTTACCGTGCCACTTCTCAGATTTGAGAAG
sgRNA-*mcr-1*	GAAAUUAAUACGACUCACUAUAGGGGUCUAGAGGACAGAAUUUUUCAACGGGUGUGCCAAUGGCCACUUUCCAGGUGGCAAAGCCCGUUGAGCUUCUCAAAUCUGAGAAGUGGCACGAUGACUUUGUCGCUGCCAA
sgRNA-*tet*(X4)	GAAAUUAAUACGACUCACUAUAGGGGUCUAGAGGACAGAAUUUUUCAACGGGUGUGCCAAUGGCCACUUUCCAGGUGGCAAAGCCCGUUGAGCUUCUCAAAUCUGAGAAGUGGCACGGUAAGUCUUGGAAAAGUAA
Probe	5′-^6^ FAM-N12-3′-BHQ1
mcr-1JF	CGGTCAGTCCGTTTGTTC
mcr-1JR	CTTGGTCGGTCTGTAGGG
tet(X4)-JF	CCGATATTCATCATCCAGAGG
tet(X4)-JR	CGCTTACTTTTCCAAGACTTACCT

The underlined nucleotides represent protospacer sequences used for CRISPR-Cas12b detection system. The remaining residuals in sgRNA-*mcr-1* and sgRNA-*tet*(X4) were necessary for Cas12b recognization

### Design and preparation of sgRNAs

In total, 36 currently known *mcr-1* variants were downloaded (Table S1) (Che et al. 2023). The 383 *tet*(X4) sequences available in the NCBI MicroBIGG-E database (as of October 20, 2022) were downloaded, and 9 sequence variants were identified using cd-hit-est with parameter -c 1 (Table S2). The *mcr-1* and *tet*(X4) sequences were aligned using MEGA X software to identify their conserved regions. The completely conserved sequence regions with a Cas12b-preferring “TTA” PAM motif were then identified in the aligned *mcr-1* and *tet*(X4) sequences, and the 20 bp protospacer sequences were extracted accordingly. Following this, the protospacer sequences were blasted against the nt database (91,700,559 sequences; total length, 1.075 Tb; database construction date, March 27, 2023) using BLASTN with the parameters “-task blastn-short, 100% identity, and 100% coverage.” The sequences containing the protospacers were then extracted from the nt database and compared with *mcr-1* or *tet*(X4) sequences to determine the specificity and conservation of the protospacers. Finally, a protospacer sequence that exhibited high conservation and specificity (1104 hits in the nt database, of which 1102 sequences were *mcr-1* positive and two were from irrelevant eukaryotic genomes, namely *Campaea margaritaria* and *Yponomeuta rorrellus*) was identified for *mcr-1* (Fig. S1A and Table 1). Similarly, a highly conserved and specific protospacer was identified for *tet*(X4) (205 hits in the nt database, of which 202 sequences were *tet*(X4) positive, one was only 93 bp long and was therefore excluded from further analysis, and two were from irrelevant eukaryotic genomes, namely *Apotomis turbidana* and *A. betuletana*) (Fig. S1B and Table 1).

The two specific protospacers for *mcr-1* and *tet*(X4) were cloned into the pUC18 vector and amplified using the mcr-sg-F and mcr-sg-R primers and tet(X4)-sg-F and tet(X4)-sg-R primers, respectively. PCR products were purified using the TaKaRa MiniBEST Agarose Gel DNA Extraction Kit (TaKaRa, Japan) and used as the template for sgRNA biosynthesis using the Transcriptaid T7 Transcription Kit (Thermo Fisher Scientific, USA) by incubating at 37 °C for 12 h. To digest the remaining DNA fragments, DNase I was added to the aforementioned reaction solution and incubated at 37°C for 30 min. Then, the transcribed sgRNA-mcr-1 and sgRNA-tet(X4) of *mcr-1* and *tet*(X4), respectively, were purified using the RNA Clean & Concentrator-5 Kit (Zymo Research, USA) and stored at − 80 °C.

### Assessment of target cleavage activity of Cas12b protein and assembly of CRISPR-Cas12b-based detection systems

The Cas12b (AacCas12b) gene was amplified from *Alicyclobacillus acidoterrestris* strain, cloned into the pET-28a vector, then transformed into *E. coli* strain BL21 (DE3). The subsequent Cas12b expression and purification processes were conducted following the methodology outlined in our prior study (Huang et al. 2021). To verify the cis-cleavage activity of the Cas12b protein, we amplified a 1000 bp region containing the specific protospacer for *mcr-1* and *tet*(X4) from *E. coli* MCE9 and *E. coli* 3934 using primer set mcr-1JF/ mcr-1JR and tet(X4)-1JF/ tet(X4)-1JR, respectively. The specific protospacer sequences were located at ~ 750 bp within the PCR products. The PCR assays commenced with an initial denaturation step at 98 °C for 3 min, followed by 30 cycles of denaturation at 98 °C for 30 s, annealing at 55 °C for 30 s, and extension at 72 °C for 1 min, and a final extension step at 72 °C for 10 min. The *mcr-1* and *tet*(X4) PCR product was mixed with Cas12b and the obtained transcribed sgRNA-mcr-1 and sgRNA-tet(X4), respectively, and the mixture was incubated at 48 °C for 30 min. Following this, 3 mL of 6 × Cas-STOP Loading Buffer was added to the mixture and incubated at 65 °C for 5 min before separation by gel electrophoresis.

To verify the trans-cleavage activity of the Cas12b protein, the CRISPR-Cas12b-based detection systems targeting *mcr-1* and *tet*(X4) were prepared in three steps. In step A, a 20 µL reaction system containing 2 µL Reaction NEBuffer 2.1 (10 ×), 0.5 µL Cas12b (250 nM), 0.5 µL sgRNA-mcr-1/sgRNA-tet(X4) (250 nM), 2 µL target DNA (should contain more than 10 copies of targets, 6.25 × 10^8^ copies for *mcr-1* and 9 × 10^8^ copies for *tet*(X4) in this study), 1 µL ssDNA-FQ probe sensor 5’- 6FAM-N12-3’-BHQ1 (500 nM), and RNase-free water was set up. In step B, the reaction system was mixed and incubated at 48 °C for 30 min and then incubated at 65 °C for 5 min. In step C, when the target DNA matched the specific protospacers of *mcr-1* and *tet*(X4), the fluorescence intensity was assessed and captured using a Dual LED Blue/White Light Transilluminator (Solarbio, China) under 485 nm light. The resulting images were saved and analyzed using ImageJ to calculate fluorescence values.

### Assessment of specificity of CRISPR-Cas12b-based detection systems

To assess the specificity of the CRISPR-Cas12b-based systems for detecting *mcr-1* and *tet*(X4), the DNA samples of *E. coli* MCE9, *E. coli* 3934, *Salmonella*, *L. monocytogenes*, and several other foodborne pathogenic bacteria were used as target templates. The DNA samples were added to the CRISPR-Cas12b-based detection systems, and the presence of a green fluorescence signal for each reaction system was assessed under 485 nm light.

### RPA primer design and reaction conditions

RPA primer pairs were designed to amplify the protospacer-containing regions of *mcr-1* and *tet*(X4). Three sets of RPA primer pairs were obtained for *mcr-1* and *tet*(X4), respectively (Table 1). To evaluate the efficacy of the designed primer pair sets, an RPA reaction was performed using the DNA Isothermal Temperature Rapid Amplification Kit (Amplification Future, China). In total, 50 µL of the reaction mixtures included 29.4 µL of buffer A solution, 2 µL of a forward primer, 2 µL of a reverse primer, 12.1 µL of nuclease-free ddH_2_O, 1 µL of genomic DNA (50 ng/µL), and 2.5 µL of buffer B added into 0.2 mL PCR tubes. The mixtures were subsequently incubated at 39°C for 30 min, followed by purification with an equal volume of phenol/chloroform and centrifugation at 12,000 rpm for 5 min. From the resulting eluate, 3 µL of the PCR product was subjected to 1% agarose gel electrophoresis for 30 min to assess its quality and purity.

### Assembly of one-tube RPA-CRISPR-Cas12b-based detection systems

While the optimal reaction temperature for RPA is 37–42 °C, Cas12b exhibits enzymatic activity within a relatively wide temperature range; therefore, we determined the reaction temperature for the one-tube RPA-CRISPR-Cas12b-based detection systems. First, a two-step approach was employed to screen the optimal amplification primers for the RPA assay (Aman et al. 2022). Specifically, for each gene, the PCR products amplified using the three sets of RPA primers were introduced into the CRISPR-Cas12b-based detection systems. The primer pair set that yielded the best performance in the aforementioned step was selected for the one-tube RPA-CRISPR-Cas12b-based detection systems. Second, the optimal reaction temperature for the one-tube assay was determined. The reaction system for the one-tube assay consisted of 14.7 µL of buffer A, 1.25 µL of buffer B, 1 µL of a forward primer, 1 µL of a reverse primer, 2 µL of genomic DNA (50 ng/µL), 2 µL of Reaction NEBuffer 2.1 (10 ×), 0.5 µL of Cas12b, 0.5 µL of sgRNA-mcr-1/sgRNA-tet(X4), 1 µL of ssDNA-FQ probe sensor, and RNase-free water. Gradient temperatures of 37 °C, 38.5 °C, 42 °C, 43.3 °C, and 45 °C were established to evaluate the performance by determining the fluorescence density values after a 1 h reaction period. The optimal primers identified using the two-step approach may not necessarily exhibit the best amplification efficiency in the one-tube detection systems (Aman et al. 2022). Hence, the one-tube detection systems were finally configured using the three primer pairs targeting *mcr-1* and *tet*(X4) at the determined optimal reaction temperatures. Their performance was evaluated based on the fluorescence values obtained at 15-min intervals over a 1-h reaction period.

### Assessment of the sensitivity of one-tube RPA-CRISPR-Cas12b-based detection systems

To assess the sensitivity of the one-tube RPA-CRISPR-Cas12b-based detection systems, the *mcr-1*- or *tet*(X4)-specific protospacer-containing pUC18 vector was diluted by 1:10 serial dilutions. Subsequently, 2 µL of the resulting solution was utilized as the DNA template in the one-tube detection systems. Following the completion of the reaction, green fluorescence was visually assessed under 485 nm light. The fluorescence density values were analyzed using ImageJ.

### Assessment of one-tube RPA-CRISPR-Cas12b-based detection systems by using pork samples spiked with *mcr-1* and *tet*(X4) contamination.

Fresh pork samples were procured from a local retail market in Yangzhou and subsequently diced into approximately 2.5 g portions. The diced samples were then treated with ultraviolet light for 45 min within a biosafety cabinet with periodic flipping every 5 min. The samples were then washed twice with ddH2O to eliminate any potential interference from pork-carried *mcr-1* and *tet*(X4) during the detection process. Following this, 2.5 mL of *E. coli* DH5M containing the *mcr-1*-carrying plasmid pUC18 or *E. coli* DH5T containing the *tet*(X4)-carrying plasmid pUC18 (OD_600_ = 1) was inoculated into 22.5 mL of LB broth to generate the initial inoculants (OD_600_ = 0.1). The inoculants were sequentially diluted by 1:10 serial dilutions. Subsequently, 2.5 g of cleaned pork samples were placed into sampling bags containing varying dilutions of bacterial solutions and incubated at 37 °C under 120 rpm for 1 h. The pork samples were then removed from the bacterial solutions, dried using sterilized filter paper, and transferred to new sampling bags containing 22.5 mL PBS solution. After homogenization for 2 min, 10 µL of bacterial solution was collected for bacterial cultivation and 1 mL of bacterial solution was used for DNA extraction. The solution was centrifuged at 8000 rpm for 5 min, and the bacterial pellet was then washed twice with PBS and resuspended in 50 µL nuclease-free water. The bacterial suspension was boiled at 100 °C for 10 min and centrifuged at 8,000 rpm for 5 min. The resulting supernatant was aspirated as a DNA template for the detection systems. The populations of *mcr-1*-positive *E. coli* DH5M and *tet*(X4)-positive *E. coli* DH5T in the contaminated pork samples were also determined using the gradient dilution plating method.

### Evaluation of one-tube RPA-CRISPR-Cas12b-based detection systems based on pork and environmental samples collected from retail markets

To evaluate the practical efficacy of our newly developed one-tube RPA-CRISPR-Cas12b-based systems for detecting *mcr-1* and *tet*(X4), a total of 40 pork samples and environmental samples (including chopping boards, workbenches, and 100-cm2 areas on the ground in the raw meat sales area) were collected from three retail markets in Yangzhou, Jiangsu, China, in May 2022. After each sample was mixed well with PBS, 1 mL of the sample was transferred into a 1.5 mL centrifuge tube and centrifuged at 8000 g for 5 min. The PBS washing step was repeated twice, followed by resuspension in 50 µL of nuclease-free water. The resulting suspension was then boiled at 100 °C for 10 min and centrifuged at 5,000 rpm for 5 min. The obtained supernatant served as the input template DNA for the one-tube RPA-CRISPR-Cas12b-based detection systems to determine whether the samples were contaminated with *mcr-1* or *tet*(X4).

Furthermore, bacterial cultivation were performed to reveal the *mcr-1* and *tet*(X4) contamination rates in the pork and environmental samples. The *mcr-1* and *tet*(X4)-specific primer sets described by Liu et al. (2016) and He et al. (2019) were used for the *mcr-1* and *tet*(X4) detection PCR assays, respectively. The DNA templates obtained from the belowmentioned colonies were used as input templates in the assays. The PCR products were verified by Sanger sequencing. Colistin- and tigecycline-resistant strains were isolated using an optimized method described by Li et al. (2022a, b, c, d, e), respectively. In brief, the collected samples were placed in centrifuge tubes containing 5 mL of peptone-buffered water and incubated at 37 °C for 24 h. Then, the bacterial suspensions were sequentially diluted by 1:10 serial dilutions. Subsequently, 100 µL of the 10 − 4 gradient was absorbed and spread on MH_2_ agar containing 2 mg/L colistin and on that containing 2 mg/L tigecycline. The plates were then incubated at 37°C for 12–16 h. Typical single colonies with inconsistent morphology were selected by colony PCR to determine the presence of *mcr-1* or *tet*(X4). The taxonomy of the identified *mcr-1*-positive and *tet*(X4)-positive strains was determined based on their 16S rDNA sequences.

Whole-genome sequencing and bioinformatics analysis of *mcr-1*- and *tet*(X4)- positive strains.

From the obtained *mcr-1*- and *tet*(X4)-positive strains, two representative strains (one *mcr-1*-positive strain and one *tet*(X4)-positive strain) were selected for whole-genome sequencing using an Illumina and Nanopore hybrid sequencing approach. In brief, genomic DNA was extracted using the QIAamp® PowerSoil® Pro Kit (Qiagen, Germany), according to the manufacturer’s protocol. The DNA concentration and integrity were assessed using 0.8% agarose gel electrophoresis, and Qubit 4.0 (Thermo Fisher Scientific, USA). High-quality long reads generated by Nanopore sequencing (Q > 7 and length > 1 kb) were assembled using Flye (v2.8.3) (Kolmogorov et al. 2019), and the assembled contigs were polished using NextPolish (v2) (Hu et al. 2020) software based on the Illumina short reads. The plasmid replicons were identified using PlasmidFinder implemented in the CGE platform based on the Enterobacteriaceae database with the following parameters: minimum 95% identity and 85% query coverage (Carattoli et al. 2014). The plasmid sequences containing *mcr-1* and *tet*(X4) were blasted against the NCBI nt database, and the closely-related plasmid sequences in the nt database were extracted and compared using BRIG (Alikhan et al. 2011). ARGs were also predicted from the *mcr-1*- and *tet*(X4)-positive plasmids using Resistance Gene Identifier (RGI, v5.1.0) based on the Comprehensive Antibiotic Resistance Database (CARD) (Alcock et al. 2020). The genomic sequences of the two isolates have been deposited in the NCBI database under the accession numbers CP126999-CP127002 for strain YZ2-2 and CP127003-CP127008 for strain YZ3-11, respectively.

## Results

### Assembly and specificity assessment of CRISPR-Cas12b-based *mcr-1* and *tet*(X4) detection systems

**Fig. 1:**
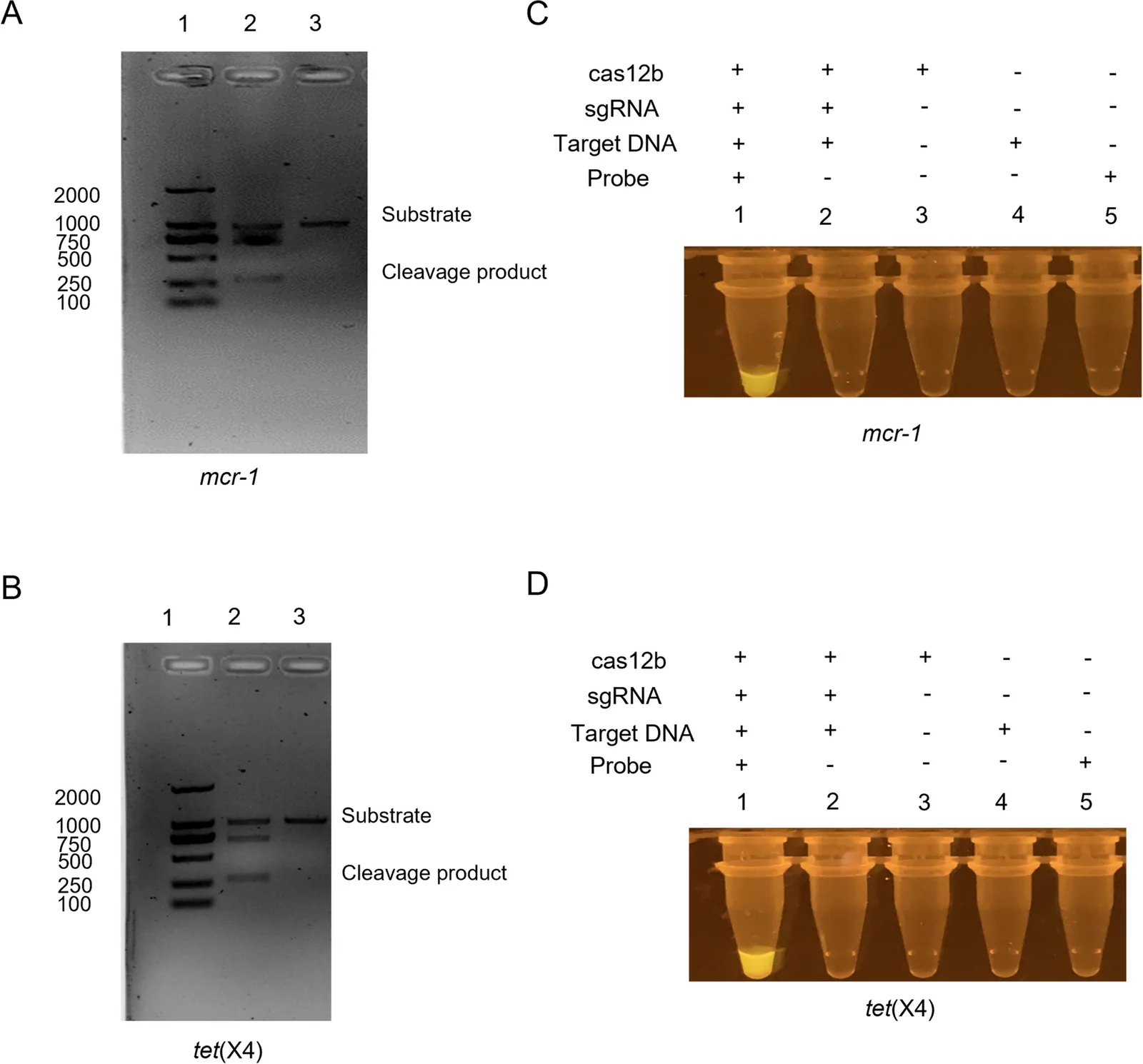
The feasibility assessment of the *cis*-cleavage activity and *trans*-cleavage activity of Cas12b to *mcr-1* (**A** and **C**) and *tet*(X4) (**B** and **D**), respectively (1, DL2000 marker; 2, Cleavage products after the Cas12b cis-cleavage reaction; 3, negative product)

The *mcr-1*- and *tet*(X4)-conserved and -specific protospacer sequences used for assembling the CRISPR-Cas12b-based detection systems were identified using a comprehensive BLAST approach. In brief, after the representative *mcr-1* and *tet*(X4) sequences were aligned, the Cas12b-preferred PAM motif “TTA” and the corresponding protospacers were identified in the conserved regions of the aligned *mcr-1* and *tet*(X4) sequences. The conversation and specificity of the identified protospacers were then determined by blasting against the NCBI nt database (see “Materials and methods”). Finally, a protospacer that was conserved in all 1042 identified *mcr-1* sequences deposited in the NCBI nt database but was only identified in two irrelevant eukaryotic genomes was used for the assembly of the CRISPR-Cas12b-based *mcr-1* detection system (Fig. S1A). Similarly, a *tet*(X4)-conserved and -specific protospacer that was conserved in 202 *tet*(X4) sequences and three non-*tet*(X4)-originating sequences identified based on the nt database was also identified. The sgRNAs were synthesized based on the two identified protospacer sequences, and the CRISPR-Cas12b-based *mcr-1* and *tet*(X4) detection systems were consequently assembled. Then, the sensitivity and specificity of the assembled detection systems were evaluated. Specifically, 1000 bp sequence amplicons of *mcr-1* and *tet*(X4) were amplified, with protospacer targets being located at 750 bp in the PCR product. Subsequently, each PCR product was incorporated into the detection systems to assess the cleavage activity of the Cas12b protein. The *mcr-1* and *tet*(X4) PCR products were successfully cleaved into two fragments with expected sizes of approximately 250 and 750 bp, respectively, indicating that sgRNAs of *mcr-1* and *tet*(X4) could effectively guide Cas12b to cis-cleave the target sequence (Fig. 1 A and B). Upon the addition of the ssDNA-FQ fluorescent probe, a robust green fluorescence signal was observed for both detection systems (Fig. 1 C and D). Collectively, these results demonstrate the feasibility of utilizing the CRISPR-Cas12b-based systems for detecting *mcr-1* and *tet*(X4).

**Fig. 2:**
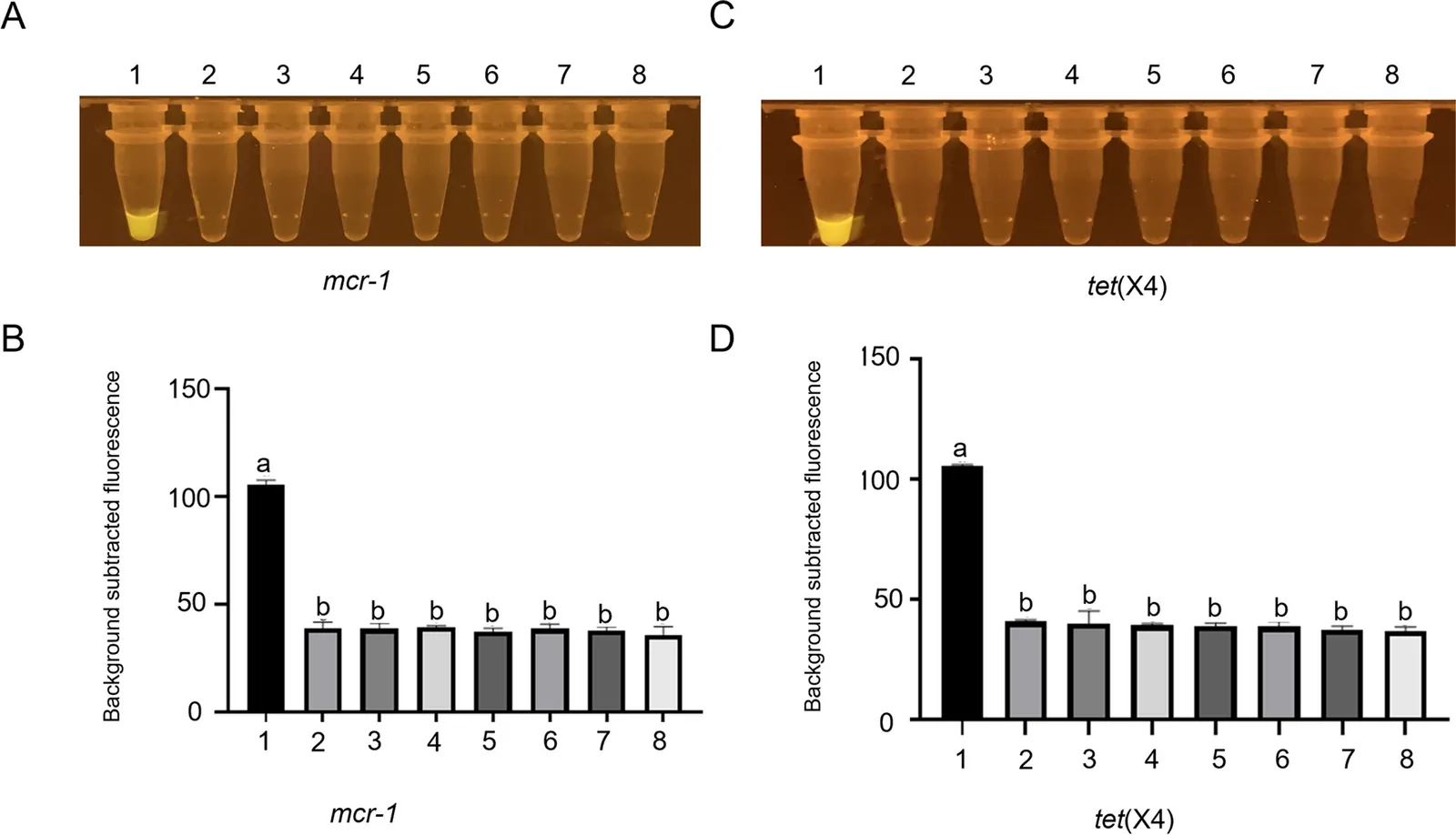
The specificity assessment and fluorescence intensity of the CRISPR-Cas12b detection system for *mcr-1* (**A** and **B**) and *tet*(X4) (**C** and **D**) detection, respectively. Different letters on the top of columns denote significant differences (*P* < 0.05, ANOVA). Error bars represent means ± SEM (*n* = 3 replicates). In **A** and **B**, sample 1 represents the *mcr-1*-positive strain *E. coli* MCE9; samples 2–7 represent several zoonotic bacterial pathogens that do not contain *mcr-1*, including *E. coli* ATCC25922, *C. jejuni* NCTC11168, *S.* Enteritidis C50041, *S.* Typhimurium SL1344, *S. aureus* ATCC27217, and *L. monocytogenes* EGD-e; and sample 8 represents negative control; in **C** and **D**, sample 1 represents the *tet*(X4)-positive strain *E. coli* MCE9; samples 2–7 represent several zoonotic bacterial pathogens that do not contain *tet*(X4), including *E. coli* ATCC25922, *C. jejuni* NCTC11168, *S.* Enteritidis C50041, *S.* Typhimurium SL1344, *S. aureus* ATCC27217, and *L. monocytogenes* EGD-e; and sample 8 represents negative control. The “background subtracted fluorescence” values were fluorescence values recorded in the reaction tubes minus the average fluorescence value obtained from three blank tubes

We then selected DNA samples from the *tet*(X4)-positive strain *E. coli* 3934, the *mcr-1*-positive strain *E. coli* MCE9, and several other zoonotic bacterial pathogens that do not contain *mcr-1* and *tet*(X4), including *E. coli* ATCC25922, *C. jejuni* NCTC11168, *S.* Enteritidis C50041, *S.* Typhimurium SL1344, *S. aureus* ATCC27217, and *L. monocytogenes* EGD-e, to evaluate the specificity of the CRISPR-Cas12b-based *mcr-1* and *tet*(X4) detection systems. The results demonstrated that only the tube containing the DNA of the *mcr-1*-positive strain *E. coli* MCE9 exhibited a strong green fluorescence signal for the *mcr-1* detection system (*P* < 0.05, ANOVA) (Fig. 2 A and B); however, no discernible green fluorescence was noted in other tubes containing DNA samples from non-*mcr-1* strains. A similar result was observed for the *tet*(X4) detection system; only the *tet*(X4)-positive strain *E. coli* 3934 could activate the system (*P* < 0.05, ANOVA) (Fig. 2 C and D). These results clearly demonstrate that the CRISPR-Cas12b-based *mcr-1* and *tet*(X4) detection systems could specifically recognize *mcr-1*- and *tet*(X4)-derived DNA in the DNA samples.

### Development of one-tube RPA-CRISPR-Cas12b-based *mcr-1* and *tet*(X4) detection systems

Previous studies have demonstrated that the combination of RPA and CRISPR-Cas-based detection systems can improve detection sensitivity and specificity (Hijjawi et al. 2023). We then tried to employ RPA in the CRISPR-Cas12b-based detection systems to enhance the efficacy of the systems for *mcr-1* and *tet*(X4) detection. The RPA primer pairs were designed based on the aligned *mcr-1* and *tet*(X4) sequences, with the protospacer sequence being located in the central part of the amplified regions. Three pairs were designed for *mcr-1* and *tet*(X4) each. All the designed primer pairs could amplify the target regions; however, the amplification efficiency varied among the primer pairs (Fig. S2). For instance, the *mcr-1* primer pair set 2 exhibited lower amplification efficiency than the primer pair sets 1 and 3 (Fig. S2A), while the *tet*(X4) primer pair set 3 produced a relatively higher concentration of PCR products than the other two primer pair sets (Fig. S2B). Because of the inconsistent amplification efficiency of each RPA primer pair, a two-step approach was employed to screen the optimal primers for *mcr-1* and *tet*(X4). For *mcr-1*, the primer pair set mcr-RPA-2 that exhibited low amplification efficiency was eliminated from further analysis (Fig. S2A). Subsequently, RPA was performed on the primer pair sets mcr-RPA-1 and mcr-RPA-3. The amplified products were then added to the CRISPR-Cas12b-based detection system. The product from the primer pair set mcr-RPA-3 could more efficiently activate the detection system and generate a more intense fluorescent signal than that from the primer pair set mcr-RPA-1 (*P* < 0.05, ANOVA) (Fig. S3A). Therefore, mcr-RPA-3 was identified as the optimal primer pair set for the RPA-CRISPR-Cas12b-based *mcr-1* detection system. The *tet*(X4) detection system containing the amplification product of the three *tet*(X4) primer pairs exhibited strong fluorescence signals. In particular, the fluorescence signal from the primer pair set tet-RPA-1 was slightly but significantly higher than that from the other two primer pair sets (*P* < 0.05, ANOVA) (Fig. S3B). Thus, tet-RPA-1 was identified as the optimal primer pair set for detecting *tet*(X4) by combining RPA and CRISPR-Cas12b.

**Fig. 3:**
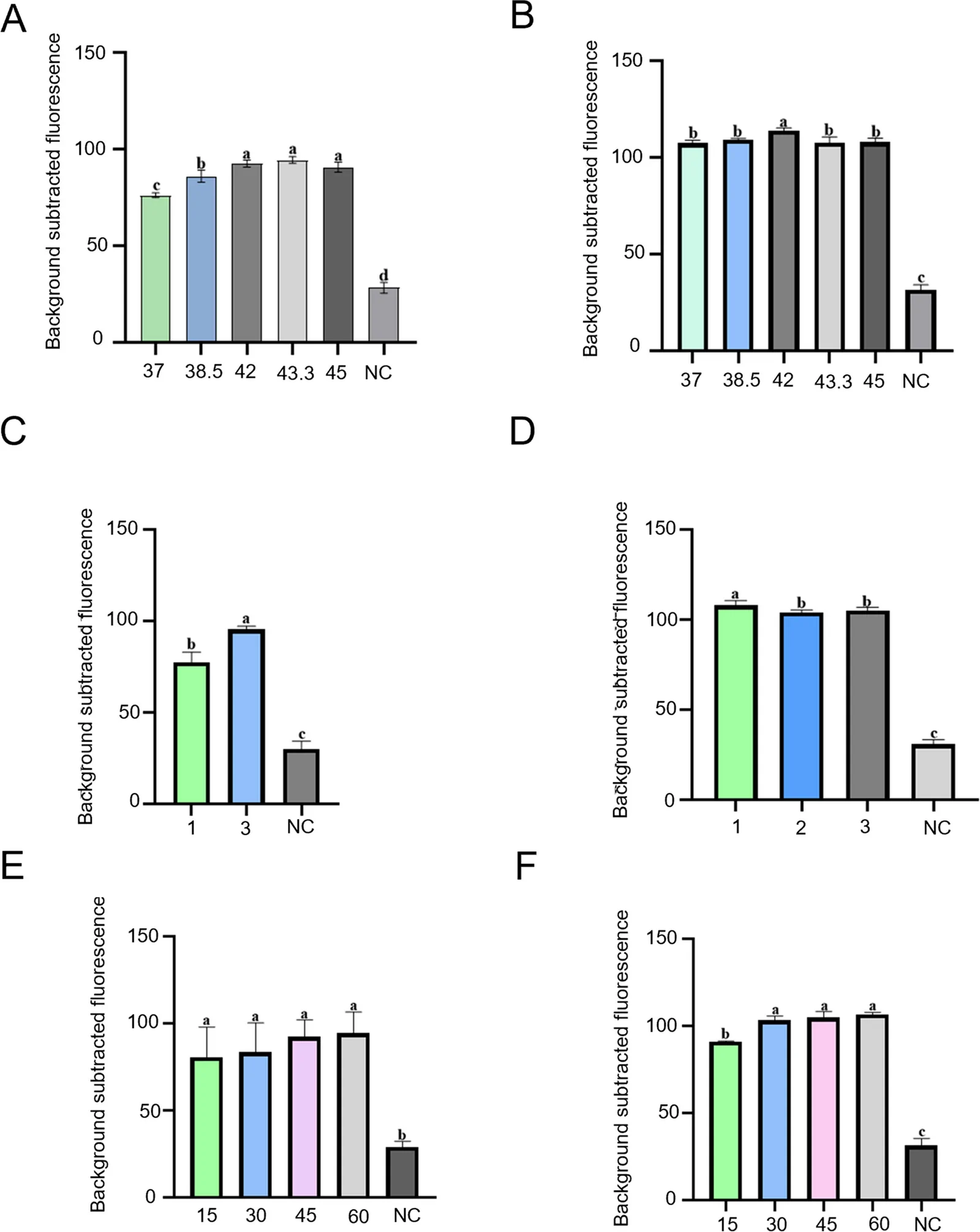
The optimal temperature determination (**A** and **B**), the optimal RPA primer set (**C** and **D**), and the optimal reaction time (**E** and **F**) for the one-tube RPA-CRISPR-Cas12b-based detection systems. Different letters on the top of columns denote significant differences (*P* < 0.05, ANOVA). Error bars represent means ± SEM (*n* = 3 replicates). The “background subtracted fluorescence” values were fluorescence values recorded in the reaction tubes minus the average fluorescence value obtained from three blank tubes

As the optimal temperature of RPA and Cas12b is different (RPA operates optimally at 37–42 °C and Cas12b at 45–55 °C), the optimal reaction temperature for the one-tube RPA-CRISPR-Cas12b-based *mcr-1* and *tet*(X4) detection systems was determined at five representative temperatures ranging from 37 to 45 °C (37 °C, 38.5 °C, 42 °C, 43.3 °C, and 45 °C). For the *mcr-1* detection system, the fluorescence density gradually increased when the temperature increased from 37 to 43.3 °C. The fluorescence density slightly decreased at 45 °C compared with that at 43.3 °C (*P* < 0.05, ANOVA) (Fig. 3A). These results suggest that the activity of Cas12b gradually augmented with an increase in temperature; however, the RPA amplification efficacy decreased when the temperature became higher than 42 ℃. Thus, the system reached its vertex at 43.3 °C, which was recognized as the optimal reaction temperature for the *mcr-1* detection system. The fluorescence signal of the *tet*(X4) detection system gradually increased as the temperature increased from 37 to 42 ℃ and then decreased as the temperature increased from 42 to 45 ℃, indicating that 42 ℃ was the optimal temperature for the *tet*(X4) detection system (*P* < 0.05, ANOVA) (Fig. 3B). As the identified optimal temperature for the one-tube RPA-CRISPR-Cas12b-based *mcr-1* and *tet*(X4) detection systems (43.3 ℃ for *mcr-1* and 42 ℃ for *tet*(X4)) differed from that for the RPA reactions in the aforementioned two-step detection system setup (39°C), the identified optimal primer pair sets for the two genes may not exhibit the best performance in the one-tube detection systems. Therefore, we evaluated the amplification efficiency of the three *mcr-1* and *tet*(X4) RPA primer pairs in the one-tube detection systems. We found that mcr-RPA-3 and tet-RPA-1 remained the optimal primer pairs for the one-tube RPA-CRISPR-Cas12b-based *mcr-1* and *tet*(X4) detection systems (*P* < 0.05, ANOVA) (Fig. 3 C and D).

We then determined the optimal reaction time for the one-tube RPA-CRISPR-Cas12b-based detection systems. As depicted in Fig. 3E, the fluorescence signal intensity of the *mcr-1* detection system gradually increased but became relatively stable from 45 min (*P* < 0.05, ANOVA). Hence, we determined that 45 min was the optimal reaction time for the one-tube *mcr-1* detection system. The fluorescence signal of the *tet*(X4) detection system was no longer significantly enhanced after 30 min, indicating that the optimal reaction time for the *tet*(X4) detection system was 30 min (*P* < 0.05, ANOVA) (Fig. 3F).

**Fig. 4:**
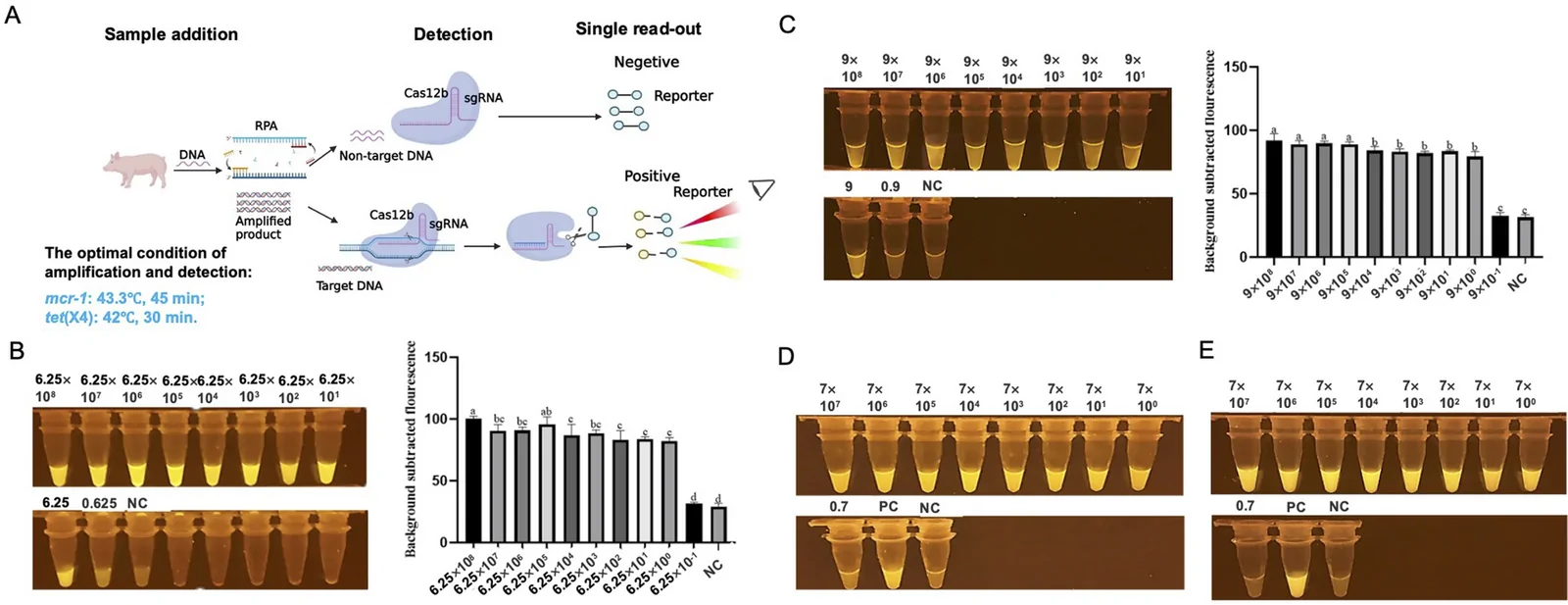
The flowchart of the one-tube RPA-CRISPR-Cas12b-based detection system (**A**); the sensitivity assessment and fluorescence intensity of the one-tube RPA-CRISPR-Cas12b-based *mcr-1* (**B**) and *tet*(X4) (**C**) detection system; the detection results of pork samples contaminated with different dose of *mcr-1* (**D**) and *tet*(X4) (**E**) positive strain, PC: positive control, NC: negative control. Different letters on the top of columns denote significant differences (*P* < 0.05, ANOVA). Error bars represent means ± SEM (*n* = 3 replicates). The “background subtracted fluorescence” values were fluorescence values recorded in the reaction tubes minus the average fluorescence value obtained from three blank tubes

Based on these optimized parameters, the one-tube RPA-CRISPR-Cas12b-based *mcr-1* and *tet*(X4) detection systems were developed (Fig. 4A).

### Assessment of sensitivity of one-tube RPA-CRISPR-Cas12b-based *mcr-1* and *tet*(X4) detection systems

We then assessed the sensitivity of the one-tube RPA-CRISPR-Cas12b-based *mcr-1* and *tet*(X4) detection systems using serially diluted DNA templates. A strong fluorescence signal was noted in tubes containing 6.25 × 10^8^ to 6.25 × 10^0^ copies of *mcr-1* for the *mcr-1* detection system; for the *tet*(X4) detection system, a strong fluorescence signal was observed in tubes containing 9 × 10^8^ to 9 × 10^0^ copies of *tet*(X4) (*P* < 0.05, ANOVA) (Fig. 4B and Fig. 4C). No obvious fluorescence signal was observed in tubes containing 6.25 × 10^−1^ copies of *mcr-1* and 9 × 10^−1^ copies of *tet*(X4). The tubes containing 1.2 copies of *mcr-1* and 1.5 copies of *tet*(X4) also did not exhibit strong fluorescence signals (Fig. S4). These results demonstrate that the capability of the one-tube RPA-CRISPR-Cas12b-based *mcr-1* detection system to achieve real-time detection with a sensitivity as low as 6.25 copies of *mcr-1*. Additionally, the sensitivity of the *tet*(X4) detection system was observed to be nine copies of *tet*(X4).

### Assessment of feasibility of one-tube RPA-CRISPR-Cas12b-based detection systems for onsite detection of *mcr-1 *and *tet*(X4).

The practicality of the one-tube RPA-CRISPR-Cas12b-based detection systems for the onsite detection of *mcr-1* and *tet*(X4) was assessed using pork samples contaminated with *E. coli* DH5M containing the *mcr-1*-carrying pUC18 plasmid and *E. coli* DH5T containing the *tet*(X4)-carrying pUC18 plasmid. Crude DNA extracted from pork samples contaminated with 7 × 10^7^ to 7 × 10^0^ CFU/g of the *mcr-1*-positive strain *E. coli* DH5M (which was determined using the gradient dilution plating method) activated the *mcr-1* detection system and produced a robust green fluorescence signal, suggesting that the detection system had a minimum detection limit of ~ 7 CFU/g of pork sample for the *mcr-1*-positive strain *E. coli* DH5M (Fig. 4D). Similarly, the feasibility of the one-tube RPA-CRISPR-Cas12b-based detection system for the onsite detection of *tet*(X4) was assessed using pork samples contaminated with tenfold gradient dilutions of *E. coli* DH5T. Strong green fluorescence signals were observed in tubes containing crude DNA extracted from pork samples contaminated with 7 × 10^7^ to 7 × 10^0^ CFU/g of *tet*(X4)-positive *E. coli* DH5T, indicating that the detection system had a minimum detection limit of ~ 7 CFU/g of pork sample for the *tet*(X4)-positive strain *E. coli* DH5T (Fig. 4E).

Finally, we used the one-tube RPA-CRISPR-Cas12b-based systems for detecting *mcr-1* and *tet*(X4) contamination in pork products sold at retail markets and in environmental samples. We found that 15% (6/40) of the collected samples were *mcr-1* positive, as revealed using the one-tube RPA-CRISPR-Cas12b-based *mcr-1* detection system. However, only three out of the six positive samples were identified to be *mcr-1* positive using the conventional cultivation-based (bacteria isolation followed by PCR amplification) method (Fig. S5). Notably, none of the samples found to be *mcr-1* negative using the one-tube RPA-CRISPR-Cas12b-based detection system showed any positive result using the cultivation-based method. Furthermore, we performed a further bacterial enrichment experiment using the three samples that were identified to be *mcr-1* positive using the RPA-CRISPR-Cas12b-based *mcr-1* detection system but negative using the cultivation-based methods and obtained *mcr-1*-positive strains for two out of the three samples. For *tet*(X4) detection, all three methods revealed consistent results. Seven out of the 40 samples were identified to be *tet*(X4) positive (Fig. S6). These results collectively suggest that the sensitivity of the one-tube RPA-CRISPR-Cas12b-based detection systems developed in this study was higher than or equivalent to that of the conventional cultivation-based methods. However, our developed systems could produce results within 1 h, which is much shorter than the duration required by the cultivation-based (> 24 h) and PCR-based (> 2 h) methods.

**Fig. 5:**
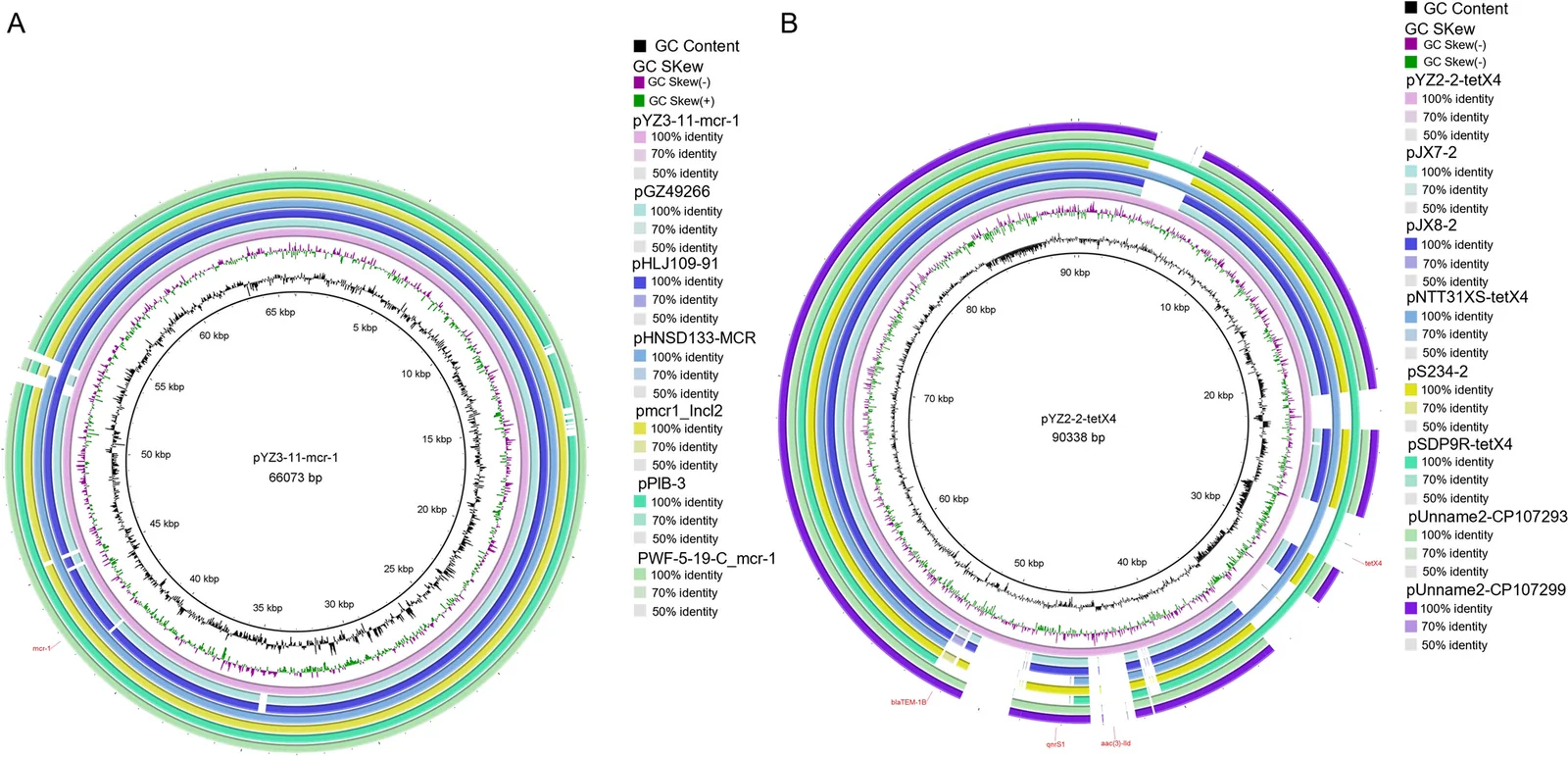
Comparisons of plasmids pYZ3-11-*mcr-1* (**A**) and pYZ2-2-*tet*X4 (**B**), and the related *mcr-1* and *tet*(X4)-carrying plasmids, respectively

We also selected an *mcr-1*-positive strain and a *tet*(X4)-positive strain isolated from the pork samples for whole-genome sequencing using Illumina short-read and Nanopore long-read technologies. The *mcr-1*-positive strain YZ3-11 was identified to be an *E. fergusonii*-affiliated strain. It was found to harbor five plasmids, and *mcr-1* was located on a 66,073 bp plasmid designated pYZ3-11-*mcr-1*, which was found to be similar to the *E. coli* plasmid that was isolated from chickens in China (including pHNSD133-MCR (NCBI accession number MG725031.1), pWF-5-19C_mcr-1 (KX505142.1), pHLJ109-91 (MN232202.1)) and the *E. coli* plasmid that isolated from humans in China (including pmcr1_IncI2 (KU761326.1), pGZ49266 (MG210938.1), pPIB-3 (CP090405.1)). Y3-11-mcr-1 is classified as an IncI2-type plasmid, which has been demonstrated to be the preferred carrier for *mcr-1* transmission among bacteria. The *tet*(X4)-positive strain YZ2-2 was identified to be a *K. pneumoniae*-affiliated strain. Three plasmids were identified in the YZ2-2 genome, and *tet*(X4) was located on a 90,338 bp plasmid designated pYZ2-2-tetX4, which was found to be similar to the *K. aerogenes* plasmids pNTT31XS-tetX4 (accession number, NZ_CP077430.1) isolated from porcine in China, the *Klebsiella* sp. plasmids pSDP9R-tetX4 (NZ_MW940621.1) isolated from pork in China, and the other five *K. pneumoniae* strains that isolated from humans in China (including pUnnamed2 (CP107293.1), pUnnamed2 (CP107299.1), pS234-2 (CP102188.1), pJX7-2 (CP064225.1), pJX8-2 (CP064219.1)) (Fig. 5B). The pYZ2-2-tetX4 plasmid is classified as an IncFII-type plasmid, which could contribute to form multiple-replicon plasmids via fusion of different single replicons, such as IncFIB, IncI1 and IncQ1, thus significantly increasing the risk of multi-drug resistance transmission (Li et al. 2021; Wang et al. 2021). In short, these results collectively indicated that there is a great risk of transmission of *mcr-1* and *tet*(X4) between humans, animals and their corresponding meat products.

## Discussion

Colistin and tigecycline are recognized as the “last line of defense” antibiotics for the treatment of infections caused by multidrug-resistant gram-negative bacteria in clinics (Lu et al. 2021; Osei et al., 2016); however, the recent emergence and quick spread of plasmid-mediated *mcr* and *tet*(X) have dramatically affected the established antibiotic therapy regimens, which could result in a lack of effective antibiotics for treating infectious diseases and compromise modern clinical medicine. Among the identified *mcr* and *tet*(X) variants, *mcr-1* and *tet*(X4) are recognized as important and prevalent gene types for colistin and tigecycline resistance, respectively (Dadashi et al. 2022; He et al. 2019). The rapid and accurate detection of *mcr-1* and *tet*(X4) is crucial for ensuring the success of antibiotic therapy in clinics and for controlling the spread and transmission of *mcr-1* and *tet*(X4) in animals and humans. In this study, we developed RPA-CRISPR-Cas-based nucleic acid detection systems for the rapid onsite detection of *mcr-1* and *tet*(X4).

The CRISPR-Cas-based nucleic acid detection system usually exhibits high specificity and sensitivity, and sgRNA is the crucial component of the detection system that guarantees its specificity (Li et al. 2022a, b, c, d, e). Therefore, the identification of a target-conserved and -specific protospacer sequence, which is the basis of sgRNA, is the crucial step for the assembly of CRISPR-Cas-based detection systems. In this study, we identified *mcr-1*- and *tet*(X4)-conserved and -specific protospacer sequences for assembling CRISPR-Cas12b-based detection systems using a comprehensive BLAST approach. The identified protospacers were highly conserved in *mcr-1* and *tet*(X4) but were only present in several irrelevant eukaryotic genomes based on the comprehensive NCBI nt database, which contains 91,700,559 sequences. The assembled CRISPR-Cas12b-based *mcr-1* and *tet*(X4) detection systems were proven to be activated only by the *mcr-1*-positive and *tet*(X4)-positive samples, respectively (Fig. 2 and S4), further demonstrating the efficacy of our bioinformatics-guided protospacer identification approach.

Notably, the CRISPR-Cas system can be combined with isothermal amplification methods, such as RPA, which offer the advantages of simplicity and rapidity, to further enhance the specificity and sensitivity of detection (Hijjawi et al. 2023; Li et al. 2022a, b, c, d, e). However, this approach involves nucleic acid amplification before detection using the CRISPR-Cas system, which may result in aerosol contamination (Lin et al. 2022). Moreover, this stepwise approach lacks convenience for onsite detection purposes. This problem can be effectively overcome by integrating nucleic acid amplification and CRISPR-Cas detection in one tube without opening the lid during the process (Lin et al. 2022). Based on this principle, we developed one-tube RPA-CRISPR-Cas12b-based detection systems that could detect as low as 6.25 copies/µL of *mcr-1* and nine copies/µL of *tet*(X4) within an hour. The conventional cultivation- and PCR-based *mcr-1* and *tet*(X4) detection methods required a sample-to-answer turnover time of > 24 h and ~ 2 h, respectively. Therefore, our developed one-tube RPA-CRISPR-Cas12b-based detection systems provided a significant advantage in terms of detection time. Furthermore, compared with other ARG detection methods, the one-tube RPA-CRISPR-Cas12b-based method exhibited a certain advantage in terms of sensitivity as well. For example, the detection levels of *mcr-1* and *tet*(X4) based on fluorescence quantitative assays were 10 copies and 102 copies, respectively (Donà et al. 2017; Li et al. 2020). We then performed *mcr-1* and *tet*(X4) detection simultaneously using our developed RPA-CRISPR-Cas12b-based detection systems and conventional cultivation-based methods to assess the prevalence of *mcr-1* and *tet*(X4) in retail pork samples and associated environmental samples. The one-tube RPA-CRISPR-Cas12b-based detection systems identified a total of six *mcr-1*-positive samples, while the conventional cultivation-based methods detected three of the six positive samples (Fig. S4). After further bacterial enrichment in the samples that were identified to be *mcr-1* positive using the one-tube RPA-CRISPR-Cas12b-based detection systems but negative using the cultivation-based method, *mcr-1*-positive strains were successfully isolated from two out of the three samples, and no *mcr-1*-positive strain was detected from sample 28 after screening 50 colonies using the colony PCR method. This discrepancy could suggest a notably lower population of *mcr-1*-positive strains in sample 28, possibly requiring a larger number of colonies for positive identification. While our extensive blast analysis indicated a low likelihood of false positives, the possibility of a false positive cannot be completely ruled out. Nevertheless, the one-tube RPA-CRISPR-Cas12b-based detection systems exhibited higher sensitivity compared with the cultivation-based method, and exhibited great potential to guide the *mcr-1-* and *tet*(X4)-positive strain isolation. However, to validate the sensitivity and specificity of the one-tube RPA-CRISPR-Cas12b-based detection systems, further large-scale sampling and detection are imperative in future studies. In addition, more analysis, encompassing the design of different *mcr-1* and *tet*(X4) sgRNAs along with exploration of varied reaction conditions, can be undertaken to enhance sensitivity and specificity while reducing the sample-to-answer time.

In addition, the high prevalence of *mcr-1* and *tet*(X4) in the pork samples and associated environmental samples revealed using the detection systems suggested that pork serves as a significant reservoir of *mcr-1* and *tet*(X4); these results are consistent with previous findings. For example, in a study on *E. coli* isolated from pork samples in Thailand, the prevalence of *mcr-1*-positive strains was as high as 56.1% (Pungpian et al. 2021). Similarly, Li et al. isolated and identified 23 *tet*(X4)-positive strains from 53 pork samples collected from markets in Jiangsu, China, and found that these strains were resistant to multiple antibiotics (Li et al. 2022a, b, c, d, e). One *mcr-1*-positive strain (YZ3-11) and one *tet*(X4)-positive strain (YZ2-2) were selected for whole-genome sequencing in our study. We found that *mcr-1* was located on the IncI2-type plasmid, which shared high similarity with the chicken-derived and human-derived plasmid isolated from China. Interestingly, this plasmid harbored only one resistance gene *mcr-1*. Moreover, *tet*(X4) was located on the IncFII-type plasmid, which exhibited high similarity with the plasmid isolated from porcine, pork, and humans in China. This result suggests that *mcr-1* and *tet*(X4) can be transmitted between humans, animals, and their corresponding meat products through the food chain.

Overall, a rapid and accurate one-tube RPA-CRISPR-Cas12b-based *mcr-1* and *tet(*X4) detection systems were developed, which exhibited exceptional specificity and sensitivity with a relatively short detection time (~ 55 min and ~ 40 min for *mcr-1* and *tet*(X4) detection, respectively). The detection systems had a detection sensitivity of 6.25 copies for *mcr-1* and nine copies for *tet*(X4). Moreover, their minimum detection limit was 7 CFU/g of pork sample for both *mcr-1*- and *tet*(X4)-positive strains. Moreover, we validated the efficacy of the detection systems using pork samples and environmental samples collected from retail markets and confirmed their application potential for the onsite detection of *mcr-1* and *tet*(X4). Overall, our developed rapid and accurate one-tube RPA-CRISPR-Cas12b-based *mcr-1* and *tet*(X4) detection systems are simple and contamination-free, require inexpensive instrumentation, and produce results within 1 h. These systems could be potentially applied to the surveillance of ARGs in the future.

## Supplementary Information

Below is the link to the electronic supplementary material.

Supplementary file1 (PDF 3017 KB)

## Data Availability

The BioProject accession number of YZ3-11 was PRJNA979119, and the accession numbers for the sequences were CP127003- CP127008; The BioProject accession number of YZ2-2 was PRJNA979126, and the accession numbers for the sequences were CP126999- CP127003.
