# Supplementary material for: Development and evaluation of rapid and accurate one-tube RPA-CRISPR-Cas12b-based detection of mcr-1 and tet(X4)
Source: Appl Microbiol Biotechnol. 2024 May 27;108:345. doi: 10.1007/s00253-024-13191-6 (PMC11129972; doi:10.1007/s00253-024-13191-6)
Supplement: Supplementary file 1 [file 253_2024_13191_MOESM1_ESM.pdf]

Applied Microbiology and Biotechnology

Development and evaluation of rapid and accurate one-tube RPA-CRISPR-Cas12b-based detection of *mcr-1* and *tet*(X4)

Yu Wang[1,2,3,4], Huan Chen[1,2,3,4], Qingyun Pan[1,2,3,4], Jing Wang[1,2,3,4], Xin'an Jiao[1,2,3,4*], Yunzeng Zhang[1,2,3,4*]

[1] Jiangsu Co-Innovation Center for Prevention and Control of Important Animal Infectious Diseases and Zoonoses, Yangzhou University, Yangzhou 225009, China

[2] Jiangsu Key Laboratory of Zoonosis, Yangzhou University, Yangzhou 225009, China

[3] Joint International Research Laboratory of Agriculture and Agri-product Safety of the Ministry of Education, Yangzhou University, Yangzhou 225009, China

[4] Key Laboratory of Prevention and Control of Biological Hazard Factors (Animal Origin) for Agrifood Safety and Quality, Ministry of Agriculture of China, Yangzhou University, Yangzhou 225009, China

*** Correspondence:**

Yunzeng Zhang, yzzhang@yzu.edu.cn; Tel: +86-5145-87971136

Address: No. 48 Wenhui East Road, College of Bioscience and Biotechnology, Yangzhou University, Yangzhou, Jiangsu, China, 225009

Xin'an Jiao, jiao@yzu.edu.cn; Tel: +86-5145-87971136

Address: No. 48 Wenhui East Road, College of Bioscience and Biotechnology, Yangzhou University, Yangzhou, Jiangsu, China, 225009

Table S1. The accession numbers of the *mcr-1* sequences used for protospacer discovery.

NG_050417.1
NG_051170.1
NG_052861.1
NG_052664.1
NG_052663.1
NG_052893.1
NG_054678.1
NG_054697.1
NG_055582.1
NG_055583.1
NG_055784.2
NG_056412.1
NG_057466.1
NG_057460.1
NG_061610.1
NG_064787.1
NG_064788.1
NG_064789.1
NG_065449.1
NG_065450.1
NG_065451.1
NG_065944.1
NG_067235.1
NG_067236.1
NG_067237.1
NG_068217.1
NG_068218.1
NG_070762.1
NG_070763.1
NG_070764.1
NG_074755.1
NG_074756.1
NG_079262.1
NG_079263.1
NG_203419.1
NG_231577.1

Table S2. The accession numbers of the *tet*(X4) sequences used for protospacer discovery.

Scientific name	Assembly	Contig	Start	Stop	Strand
Salmonella enterica subsp. *enterica* serovar Agona	GCA_004183755.1	AAABRY010000064.1	1116	2273	+
Salmonella enterica subsp. *enterica* serovar Agona	GCA_004183755.1	AAABRY010000091.1	1147	2304	+
Salmonella enterica subsp. *enterica* serovar Stanley	GCA_007249975.1	AAHLAN010000051.1	1006	2163	+
Salmonella enterica subsp. *enterica* serovar Livingstone	GCA_007264485.1	AAHMBT010000084.1	1064	2221	+
Salmonella enterica subsp. *enterica* serovar Livingstone	GCA_007264485.1	AAHMBT010000116.1	1095	2252	+
Salmonella enterica	GCA_008167565.1	AAJTXD010000043.1	1382	2539	+
Salmonella enterica	GCA_008167565.1	AAJTXD010000071.1	1383	2540	+
Salmonella enterica subsp. *enterica* serovar Typhimurium	GCA_008201095.1	AAJWIR010000186.1	1093	2250	+
Salmonella enterica subsp. *enterica* serovar Typhimurium	GCA_008201095.1	AAJWIR010000250.1	1124	2281	+
Salmonella enterica subsp. *enterica* serovar Typhimurium	GCA_008540555.1	AAKSZD010000064.1	1108	2265	+
Salmonella enterica subsp. *enterica* serovar Typhimurium	GCA_008540555.1	AAKSZD010000121.1	1135	2292	+
Salmonella enterica subsp. *enterica* serovar Agona	GCA_008576375.1	AAKUGF010000071.1	1383	2540	+
Salmonella enterica subsp. *enterica* serovar Agona	GCA_008576375.1	AAKUGF010000103.1	1062	2219	+
Salmonella enterica subsp. *enterica* serovar Kentucky	GCA_010931195.1	AAPAUS010000037.1	1619	2776	+
Salmonella enterica subsp. *enterica* serovar Kentucky	GCA_010931195.1	AAPAUS010000054.1	1384	2541	+
Salmonella enterica subsp. *enterica* serovar Stanley	GCA_011326515.1	AAPUUC010000047.1	1101	2258	+
Salmonella enterica subsp. *enterica* serovar Stanley	GCA_011326515.1	AAPUUC010000091.1	1132	2289	+
Shigella sonnei	GCA_013458775.1	AATWGR010000223.1	1102	2259	+
Shigella sonnei	GCA_013458775.1	AATWGR010000452.1	889	2046	+
Salmonella enterica	GCA_017796145.1	AAZUQE010000053.1	1042	2199	+
Salmonella enterica	GCA_017796145.1	AAZUQE010000079.1	1073	2230	+
Salmonella enterica	GCA_017802095.1	AAZVAB010000035.1	1107	2264	+
Salmonella enterica	GCA_017802095.1	AAZVAB010000064.1	1136	2293	+
Salmonella enterica	GCA_017834535.1	AAZWSE010000064.1	1066	2223	+
Salmonella enterica	GCA_017834535.1	AAZWSE010000096.1	1097	2254	+
Salmonella enterica	GCA_017865385.1	AAZXRY010000094.1	1100	2257	+
Salmonella enterica	GCA_017865385.1	AAZXRY010000137.1	1133	2290	+
Salmonella enterica subsp. *enterica* serovar Kentucky	GCA_018978845.1	ABAYCX010000037.1	1619	2776	+
Salmonella enterica subsp. *enterica* serovar Kentucky	GCA_018978845.1	ABAYCX010000053.1	1424	2581	+
Escherichia coli	GCA_022421445.1	ABFGCA010000106.1	1063	2220	+
Escherichia coli	GCA_022421445.1	ABFGCA010000156.1	1094	2251	+
Escherichia coli	GCA_022437005.1	ABFGOC010000102.1	1123	2280	+
Escherichia coli	GCA_022437005.1	ABFGOC010000162.1	345	1502	+
Escherichia coli	GCA_022633235.1	ABFLGQ010000206.1	1113	2270	+
Escherichia coli	GCA_022633235.1	ABFLGQ010000343.1	1089	2246	+
Escherichia coli	GCA_022636115.1	ABFLMC010000133.1	1069	2226	+
Escherichia coli	GCA_022636115.1	ABFLMC010000181.1	905	2062	+
Escherichia coli	GCA_022947075.1	ABFSTS010000089.1	1619	2776	+
Escherichia coli	GCA_022947075.1	ABFSTS010000123.1	1424	2581	+
Escherichia coli	GCA_023310495.1	ABGCRY010000102.1	1383	2540	+
Escherichia coli	GCA_023310495.1	ABGCRY010000157.1	1424	2581	+
Escherichia coli	GCA_023310715.1	ABGCSG010000094.1	1619	2776	+
Escherichia coli	GCA_023310715.1	ABGCSG010000142.1	1424	2581	+
Escherichia coli	GCA_023403425.1	ABGFKC010000078.1	1304	2461	+
Escherichia coli	GCA_023403425.1	ABGFKC010000149.1	1345	2502	+
Escherichia coli	GCA_025027415.1	ABIBFN010000144.1	1383	2540	+
Escherichia coli	GCA_025027415.1	ABIBFN010000271.1	1424	2581	+
Salmonella enterica	GCA_026040715.1	ABJEAM010000037.1	1946	3103	+
Salmonella enterica	GCA_026040715.1	ABJEAM010000056.1	1424	2581	+
Salmonella enterica	GCA_031661075.1	ABMPIU010000019.1	1619	2776	+
Salmonella enterica	GCA_031661075.1	ABMPIU010000035.1	1424	2581	+
Escherichia coli	GCA_918364955.1	CAKKEC010000080.1	7094	8248	-

Escherichia coli	GCA_021398675.1	CP090284.1	53354	54508	+
Escherichia coli	GCA_023099705.2	CP101799.1	34654	35808	-
Escherichia coli	GCA_023098635.2	CP101801.1	53354	54508	+
Escherichia coli	GCA_012297045.1	DAAZEY010000048.1	1383	2540	+
Escherichia coli	GCA_012297045.1	DAAZEY010000090.1	897	2054	+
Escherichia coli	GCA_012502375.1	DABAJL010000078.1	1383	2540	+
Escherichia coli	GCA_012502375.1	DABAJL010000115.1	1384	2541	+
Escherichia coli	GCA_012501995.1	DABAJO010000069.1	1403	2560	+
Escherichia coli	GCA_012501995.1	DABAJO010000097.1	1404	2561	+
Escherichia coli	GCA_013077765.1	DABIHN010000105.1	1637	2794	+
Escherichia coli	GCA_013077765.1	DABIHN010000171.1	1404	2561	+
Escherichia coli	GCA_013077305.1	DABIID010000063.1	1403	2560	+
Escherichia coli	GCA_013077305.1	DABIID010000102.1	1404	2561	+
Escherichia coli	GCA_013077385.1	DABIIH010000081.1	1383	2540	+
Escherichia coli	GCA_013077385.1	DABIIH010000119.1	1384	2541	+
Escherichia coli	GCA_013077405.1	DABIII010000081.1	1383	2540	+
Escherichia coli	GCA_013077405.1	DABIII010000118.1	1384	2541	+
Escherichia coli	GCA_014657275.1	DABTSW010000162.1	1044	2201	+
Escherichia coli	GCA_014657275.1	DABTSW010000311.1	1143	2300	+
Escherichia coli	GCA_017044295.1	DADHZV010000092.1	1382	2539	+
Escherichia coli	GCA_017044295.1	DADHZV010000194.1	1423	2580	+
Escherichia coli	GCA_017044335.1	DADHZW010000055.1	1382	2539	+
Escherichia coli	GCA_017044335.1	DADHZW010000101.1	1423	2580	+
Escherichia coli	GCA_017044315.1	DADHZX010000055.1	1382	2539	+
Escherichia coli	GCA_017044315.1	DADHZX010000108.1	1423	2580	+
Escherichia coli	GCA_017044255.1	DADHZZ010000066.1	1382	2539	+
Escherichia coli	GCA_017044255.1	DADHZZ010000102.1	1423	2580	+
Escherichia coli	GCA_017044615.1	DADIAC010000054.1	1382	2539	+
Escherichia coli	GCA_017044615.1	DADIAC010000108.1	1423	2580	+
Escherichia coli	GCA_017044575.1	DADIAD010000107.1	1382	2539	+
Escherichia coli	GCA_017044575.1	DADIAD010000195.1	1423	2580	+
Escherichia coli	GCA_017044475.1	DADIAF010000058.1	1382	2539	+
Escherichia coli	GCA_017044475.1	DADIAF010000098.1	1423	2580	+
Escherichia coli	GCA_017044535.1	DADIAH010000069.1	1382	2539	+
Escherichia coli	GCA_017044535.1	DADIAH010000132.1	1423	2580	+
Escherichia coli	GCA_017044895.1	DADIAS010000040.1	1382	2539	+
Escherichia coli	GCA_017044895.1	DADIAS010000076.1	1423	2580	+
Escherichia coli	GCA_017045015.1	DADIAT010000100.1	1382	2539	+
Escherichia coli	GCA_017045015.1	DADIAT010000176.1	1423	2580	+
Escherichia coli	GCA_017044875.1	DADIAW010000069.1	1382	2539	+
Escherichia coli	GCA_017044875.1	DADIAW010000129.1	1423	2580	+
Escherichia coli	GCA_017045215.1	DADIBC010000067.1	1382	2539	+
Escherichia coli	GCA_017045215.1	DADIBC010000134.1	1423	2580	+
Escherichia coli	GCA_017045435.1	DADIBL010000062.1	1382	2539	+
Escherichia coli	GCA_017045435.1	DADIBL010000107.1	1423	2580	+
Escherichia coli	GCA_017045275.1	DADIBR010000060.1	1382	2539	+
Escherichia coli	GCA_017045275.1	DADIBR010000116.1	1423	2580	+
Escherichia coli	GCA_017045795.1	DADICO010000040.1	1570	2727	+
Escherichia coli	GCA_017045795.1	DADICO010000077.1	1423	2580	+
Escherichia coli	GCA_017046315.1	DADIDJ010000078.1	1382	2539	+
Escherichia coli	GCA_017046315.1	DADIDJ010000172.1	1423	2580	+
Escherichia coli	GCA_017046395.1	DADIDK010000104.1	1382	2539	+
Escherichia coli	GCA_017046395.1	DADIDK010000177.1	1423	2580	+
Escherichia coli	GCA_017046635.1	DADIEF010000033.1	1382	2539	+

Escherichia coli	GCA_017046635.1	DADIEF010000078.1	1423	2580	+
Escherichia coli	GCA_017046555.1	DADIEH010000094.1	1382	2539	+
Escherichia coli	GCA_017046555.1	DADIEH010000171.1	1423	2580	+
Escherichia coli	GCA_017046695.1	DADIEK010000097.1	1382	2539	+
Escherichia coli	GCA_017046695.1	DADIEK010000219.1	1423	2580	+
Escherichia coli	GCA_017046955.1	DADIEP010000050.1	1382	2539	+
Escherichia coli	GCA_017046955.1	DADIEP010000093.1	1423	2580	+
Escherichia coli	GCA_017047015.1	DADIEY010000089.1	1382	2539	+
Escherichia coli	GCA_017047015.1	DADIEY010000152.1	1423	2580	+
Escherichia coli	GCA_017046895.1	DADIFD010000068.1	1382	2539	+
Escherichia coli	GCA_017046895.1	DADIFD010000112.1	1423	2580	+
Escherichia coli	GCA_017047535.1	DADIFO010000058.1	1382	2539	+
Escherichia coli	GCA_017047535.1	DADIFO010000109.1	1423	2580	+
Escherichia coli	GCA_017047455.1	DADIFR010000124.1	1382	2539	+
Escherichia coli	GCA_017047455.1	DADIFR010000254.1	1423	2580	+
Escherichia coli	GCA_017910375.1	DADVHM010000295.1	220	1377	-
Escherichia coli	GCA_017910375.1	DADVHM010000442.1	889	2046	+
Escherichia coli	GCA_018022355.1	DADWPG010000048.1	1382	2539	+
Escherichia coli	GCA_018022355.1	DADWPG010000094.1	1423	2580	+
Escherichia coli	GCA_018295365.1	DAEAWD010000085.1	1384	2541	+
Escherichia coli	GCA_018295365.1	DAEAWD010000128.1	906	2063	+
Escherichia coli	GCA_018295395.1	DAEAWE010000104.1	1384	2541	+
Escherichia coli	GCA_018295395.1	DAEAWE010000161.1	936	2093	+
Escherichia coli	GCA_018295325.1	DAEAWF010000046.1	1195	2352	+
Escherichia coli	GCA_018295325.1	DAEAWF010000089.1	1168	2325	+
Escherichia coli	GCA_018295385.1	DAEAWH010000104.1	1403	2560	+
Escherichia coli	GCA_018295385.1	DAEAWH010000153.1	1444	2601	+
Escherichia coli	GCA_018295425.1	DAEAWK010000102.1	1129	2286	+
Escherichia coli	GCA_018295425.1	DAEAWK010000177.1	1170	2327	+
Escherichia coli	GCA_018295465.1	DAEAWN010000053.1	1384	2541	+
Escherichia coli	GCA_018295465.1	DAEAWN010000079.1	1425	2582	+
Escherichia coli	GCA_018295445.1	DAEAWO010000093.1	1096	2253	+
Escherichia coli	GCA_018295445.1	DAEAWO010000147.1	1136	2293	+
Escherichia coli	GCA_018295525.1	DAEAWR010000104.1	1383	2540	+
Escherichia coli	GCA_018295525.1	DAEAWR010000153.1	1424	2581	+
Escherichia coli	GCA_018295535.1	DAEAWS010000121.1	1496	2653	+
Escherichia coli	GCA_018295535.1	DAEAWS010000200.1	1132	2289	+
Escherichia coli	GCA_018295565.1	DAEAWU010000038.1	1129	2286	+
Escherichia coli	GCA_018295565.1	DAEAWU010000103.1	1169	2326	+
Escherichia coli	GCA_018295645.1	DAEAWV010000134.1	1129	2286	+
Escherichia coli	GCA_018295645.1	DAEAWV010000201.1	1170	2327	+
Escherichia coli	GCA_018295585.1	DAEAWW010000081.1	1129	2286	+
Escherichia coli	GCA_018295585.1	DAEAWW010000118.1	1169	2326	+
Escherichia coli	GCA_018295865.1	DAEAWX010000072.1	1129	2286	+
Escherichia coli	GCA_018295865.1	DAEAWX010000139.1	936	2093	+
Escherichia coli	GCA_018295805.1	DAEAWY010000096.1	1094	2251	+
Escherichia coli	GCA_018295805.1	DAEAWY010000139.1	1135	2292	+
Escherichia coli	GCA_018295505.1	DAEAWZ010000080.1	1403	2560	+
Escherichia coli	GCA_018295505.1	DAEAWZ010000133.1	1444	2601	+
Escherichia coli	GCA_018295825.1	DAEAXA010000115.1	1095	2252	+
Escherichia coli	GCA_018295825.1	DAEAXA010000173.1	936	2093	+
Escherichia coli	GCA_018295665.1	DAEAXB010000073.1	1490	2647	+
Escherichia coli	GCA_018295665.1	DAEAXB010000141.1	1169	2326	+
Escherichia coli	GCA_018295685.1	DAEAXC010000051.1	1128	2285	+

Escherichia coli	GCA_018295685.1	DAEAXC010000099.1	1169	2326	+
Escherichia coli	GCA_018295765.1	DAEAXD010000070.1	1383	2540	+
Escherichia coli	GCA_018295765.1	DAEAXD010000127.1	1424	2581	+
Escherichia coli	GCA_018295605.1	DAEAXE010000052.1	1384	2541	+
Escherichia coli	GCA_018295605.1	DAEAXE010000144.1	906	2063	+
Escherichia coli	GCA_018295705.1	DAEAXF010000096.1	1096	2253	+
Escherichia coli	GCA_018295705.1	DAEAXF010000154.1	1136	2293	+
Escherichia coli	GCA_018295745.1	DAEAXG010000102.1	1383	2540	+
Escherichia coli	GCA_018295745.1	DAEAXG010000154.1	1424	2581	+
Escherichia coli	GCA_018295785.1	DAEAXH010000097.1	1094	2251	+
Escherichia coli	GCA_018295785.1	DAEAXH010000142.1	1135	2292	+
Escherichia coli	GCA_018295725.1	DAEAXI010000075.1	1128	2285	+
Escherichia coli	GCA_018295725.1	DAEAXI010000113.1	1169	2326	+
Escherichia coli	GCA_018295625.1	DAEAXJ010000097.1	1095	2252	+
Escherichia coli	GCA_018295625.1	DAEAXJ010000149.1	1136	2293	+
Escherichia coli	GCA_021088305.1	DAFGWP010000098.1	1383	2540	+
Escherichia coli	GCA_021088305.1	DAFGWP010000181.1	1424	2581	+
Escherichia coli	GCA_021453045.1	DAFLOI010000158.1	1379	2536	+
Escherichia coli	GCA_021453045.1	DAFLOI010000247.1	1420	2577	+
Escherichia coli	GCA_021454425.1	DAFLQZ010000159.1	1379	2536	+
Escherichia coli	GCA_021454425.1	DAFLQZ010000248.1	1420	2577	+
Klebsiella pneumoniae	GCA_021933115.1	DAFXYV010000074.1	1075	2232	+
Klebsiella pneumoniae	GCA_021933115.1	DAFXYV010000169.1	1114	2271	+
Klebsiella pneumoniae	GCA_022026825.1	DAGCMU010000057.1	1383	2540	+
Klebsiella pneumoniae	GCA_022026825.1	DAGCMU010000107.1	1424	2581	+
Klebsiella pneumoniae	GCA_022026775.1	DAGCMV010000058.1	1382	2539	+
Klebsiella pneumoniae	GCA_022026775.1	DAGCMV010000108.1	1423	2580	+
Klebsiella pneumoniae	GCA_022355205.1	DAGTSU010000032.1	1619	2776	+
Klebsiella pneumoniae	GCA_022355205.1	DAGTSU010000057.1	1384	2541	+
Escherichia coli	GCA_023465755.1	DAHEXP010000104.1	1040	2197	+
Escherichia coli	GCA_023465755.1	DAHEXP010000157.1	26	1183	+
Escherichia coli	GCA_023674145.1	DAHJKX010000211.1	220	1377	-
Escherichia coli	GCA_023674145.1	DAHJKX010000369.1	889	2046	+
Escherichia coli	GCA_023778665.1	DAHKSM010000062.1	1633	2790	+
Escherichia coli	GCA_023778665.1	DAHKSM010000103.1	1674	2831	+
Escherichia coli	GCA_023779205.1	DAHKTC010000118.1	1383	2540	+
Escherichia coli	GCA_023779205.1	DAHKTC010000165.1	1424	2581	+
Escherichia coli	GCA_023783615.1	DAHKXS010000080.1	1403	2560	+
Escherichia coli	GCA_023783615.1	DAHKXS010000131.1	1444	2601	+
Escherichia coli	GCA_024611375.1	DAILGF010000075.1	1402	2559	+
Escherichia coli	GCA_024611375.1	DAILGF010000127.1	1443	2600	+
Escherichia coli	GCA_024612605.1	DAILHC010000077.1	1381	2538	+
Escherichia coli	GCA_024612605.1	DAILHC010000155.1	1422	2579	+
Escherichia coli	GCA_024612725.1	DAILHH010000120.1	1402	2559	+
Escherichia coli	GCA_024612725.1	DAILHH010000261.1	1443	2600	+
Escherichia coli	GCA_024613245.1	DAILIE010000146.1	1097	2254	+
Escherichia coli	GCA_024613245.1	DAILIE010000299.1	1137	2294	+
Escherichia coli	GCA_024613445.1	DAILIK010000137.1	1252	2409	+
Escherichia coli	GCA_024613745.1	DAILJA010000093.1	1402	2559	+
Escherichia coli	GCA_024613745.1	DAILJA010000140.1	1443	2600	+
Escherichia coli	GCA_024614025.1	DAILJL010000139.1	1213	2370	+
Escherichia coli	GCA_024614025.1	DAILJL010000275.1	1138	2295	+
Escherichia coli	GCA_024614045.1	DAILJO010000062.1	1402	2559	+
Escherichia coli	GCA_024614045.1	DAILJO010000115.1	1443	2600	+

Escherichia coli	GCA_024614465.1	DAILKD010000034.1	1129	2286	+
Escherichia coli	GCA_024614465.1	DAILKD010000074.1	1169	2326	+
Escherichia coli	GCA_024614745.1	DAILKQ010000093.1	1402	2559	+
Escherichia coli	GCA_024614745.1	DAILKQ010000149.1	1443	2600	+
Escherichia coli	GCA_024674005.1	DAIUSA010000034.1	1129	2286	+
Escherichia coli	GCA_024674005.1	DAIUSA010000075.1	1169	2326	+
Escherichia coli	GCA_024674155.1	DAIUSF010000094.1	1403	2560	+
Escherichia coli	GCA_024674155.1	DAIUSF010000148.1	1444	2601	+
Escherichia coli	GCA_024674175.1	DAIUSI010000118.1	1403	2560	+
Escherichia coli	GCA_024674175.1	DAIUSI010000163.1	1444	2601	+
Escherichia coli	GCA_024674585.1	DAIUTA010000379.1	1443	2600	+
Escherichia coli	GCA_024674565.1	DAIUTC010000108.1	1402	2559	+
Escherichia coli	GCA_024674565.1	DAIUTC010000171.1	1443	2600	+
Escherichia coli	GCA_024674645.1	DAIUTE010000088.1	1402	2559	+
Escherichia coli	GCA_024674645.1	DAIUTE010000149.1	1443	2600	+
Escherichia coli	GCA_024674665.1	DAIUTG010000115.1	1402	2559	+
Escherichia coli	GCA_024674665.1	DAIUTG010000170.1	1443	2600	+
Escherichia coli	GCA_024674955.1	DAIUTT010000088.1	1403	2560	+
Escherichia coli	GCA_024674955.1	DAIUTT010000151.1	1444	2601	+
Escherichia coli	GCA_024675295.1	DAIUUK010000079.1	1435	2592	+
Escherichia coli	GCA_024675295.1	DAIUUK010000142.1	1476	2633	+
Escherichia coli	GCA_024676435.1	DAIUWL010000121.1	1403	2560	+
Escherichia coli	GCA_024676435.1	DAIUWL010000197.1	1444	2601	+
Escherichia coli	GCA_024676515.1	DAIUWO010000111.1	1403	2560	+
Escherichia coli	GCA_024676515.1	DAIUWO010000208.1	1444	2601	+
Escherichia coli	GCA_024676535.1	DAIUWR010000060.1	1401	2558	+
Escherichia coli	GCA_024676535.1	DAIUWR010000112.1	1442	2599	+
Escherichia coli	GCA_024677385.1	DAIUXD010000085.1	1383	2540	+
Escherichia coli	GCA_024677385.1	DAIUXD010000128.1	1424	2581	+
Escherichia coli	GCA_024679815.1	DAIUXX010000109.1	1436	2593	+
Escherichia coli	GCA_024679815.1	DAIUXX010000146.1	1477	2634	+
Escherichia coli	GCA_024679845.1	DAIUXZ010000069.1	1402	2559	+
Escherichia coli	GCA_024679845.1	DAIUXZ010000105.1	1443	2600	+
Escherichia coli	GCA_024682715.1	DAIUZT010000037.1	1129	2286	+
Escherichia coli	GCA_024682715.1	DAIUZT010000107.1	1169	2326	+
Escherichia coli	GCA_024684575.1	DAIVBA010000093.1	1435	2592	+
Escherichia coli	GCA_024684575.1	DAIVBA010000142.1	1476	2633	+
Escherichia coli	GCA_024686515.1	DAIVCK010000110.1	1436	2593	+
Escherichia coli	GCA_024686515.1	DAIVCK010000147.1	1477	2634	+
Escherichia coli	GCA_024686735.1	DAIVCY010000038.1	3574	4731	+
Escherichia coli	GCA_024686735.1	DAIVCY010000087.1	1116	2273	+
Escherichia coli	GCA_024687425.1	DAIVDP010000090.1	1403	2560	+
Escherichia coli	GCA_024687425.1	DAIVDP010000146.1	1444	2601	+
Escherichia coli	GCA_024687365.1	DAIVDS010000089.1	1403	2560	+
Escherichia coli	GCA_024687365.1	DAIVDS010000144.1	1444	2601	+
Klebsiella aerogenes	GCA_025094595.1	DAJDBL010000038.1	1383	2540	+
Klebsiella aerogenes	GCA_025094595.1	DAJDBL010000074.1	1424	2581	+
Klebsiella pneumoniae	GCA_025095235.1	DAJDCD010000068.1	1382	2539	+
Klebsiella pneumoniae	GCA_025095235.1	DAJDCD010000131.1	1423	2580	+
Klebsiella pneumoniae	GCA_025102265.1	DAJDGO010000057.1	1383	2540	+
Klebsiella pneumoniae	GCA_025102265.1	DAJDGO010000093.1	1424	2581	+
Escherichia coli	GCA_025328385.1	DAJIBJ010000093.1	1117	2274	+
Escherichia coli	GCA_025328385.1	DAJIBJ010000144.1	1144	2301	+
Escherichia coli	GCA_025671685.1	DAJNZE010000112.1	937	2094	+

Escherichia coli	GCA_025671685.1	DAJNZE010000182.1	964	2121	+
Escherichia coli	GCA_025671745.1	DAJNZF010000091.1	1372	2529	+
Escherichia coli	GCA_025671745.1	DAJNZF010000135.1	277	1434	+
Escherichia coli	GCA_025672085.1	DAJNZW010000090.1	904	2061	+
Escherichia coli	GCA_025672085.1	DAJNZW010000167.1	1423	2580	+
Escherichia coli	GCA_025672125.1	DAJNZX010000107.1	1065	2222	+
Escherichia coli	GCA_025672125.1	DAJNZX010000180.1	992	2149	+
Escherichia coli	GCA_025672165.1	DAJOAA010000057.1	1058	2215	+
Escherichia coli	GCA_025672165.1	DAJOAA010000111.1	1089	2246	+
Escherichia coli	GCA_025672205.1	DAJOAC010000090.1	1382	2539	+
Escherichia coli	GCA_025672205.1	DAJOAC010000123.1	1423	2580	+
Escherichia coli	GCA_025672325.1	DAJOAJ010000093.1	979	2136	+
Escherichia coli	GCA_025672325.1	DAJOAJ010000179.1	1007	2164	+
Escherichia coli	GCA_025672505.1	DAJOAR010000140.1	1020	2177	+
Escherichia coli	GCA_025672505.1	DAJOAR010000205.1	1118	2275	+
Escherichia coli	GCA_025674805.1	DAJOBW010000081.1	909	2066	+
Escherichia coli	GCA_025674805.1	DAJOBW010000147.1	1089	2246	+
Escherichia coli	GCA_025675265.1	DAJOCG010000065.1	1382	2539	+
Escherichia coli	GCA_025675265.1	DAJOCG010000118.1	1423	2580	+
Escherichia coli	GCA_025675245.1	DAJOCJ010000073.1	1114	2271	+
Escherichia coli	GCA_025675245.1	DAJOCJ010000144.1	1145	2302	+
Escherichia coli	GCA_025877765.1	DAJQNA010000175.1	1002	2159	+
Escherichia coli	GCA_025877765.1	DAJQNA010000254.1	1003	2160	+
Escherichia coli	GCA_026314675.1	DAJZWD010000090.1	1381	2538	+
Escherichia coli	GCA_026314675.1	DAJZWD010000170.1	1422	2579	+
Escherichia coli	GCA_026348895.1	DAKBWI010000090.1	1381	2538	+
Escherichia coli	GCA_026348895.1	DAKBWI010000179.1	1348	2505	+
Escherichia coli	GCA_026348915.1	DAKBWK010000070.1	1383	2540	+
Escherichia coli	GCA_026348915.1	DAKBWK010000120.1	1424	2581	+
Escherichia coli	GCA_026348995.1	DAKBWM010000082.1	973	2130	+
Escherichia coli	GCA_026348995.1	DAKBWM010000136.1	1012	2169	+
Escherichia coli	GCA_027361615.1	DALSPI010000072.1	1403	2560	+
Escherichia coli	GCA_027361615.1	DALSPI010000137.1	21	1178	+
Escherichia coli	GCA_028829915.1	DANMJS010000045.1	1491	2648	+
Escherichia coli	GCA_028829915.1	DANMJS010000096.1	1170	2327	+
Escherichia coli	GCA_028830155.1	DANMJT010000094.1	1098	2255	+
Escherichia coli	GCA_028830155.1	DANMJT010000159.1	1136	2293	+
Escherichia coli	GCA_028830425.1	DANMKC010000064.1	1279	2436	+
Escherichia coli	GCA_028830425.1	DANMKC010000144.1	1166	2323	+
Escherichia coli	GCA_028830365.1	DANMKD010000122.1	1403	2560	+
Escherichia coli	GCA_028830365.1	DANMKD010000178.1	1444	2601	+
Escherichia coli	GCA_028830215.1	DANMKE010000068.1	1098	2255	+
Escherichia coli	GCA_028830215.1	DANMKE010000128.1	1138	2295	+
Escherichia coli	GCA_028830235.1	DANMKF010000120.1	1403	2560	+
Escherichia coli	GCA_028830235.1	DANMKF010000179.1	1444	2601	+
Escherichia coli	GCA_028830235.1	DANMKF010000180.1	1444	2601	+
Escherichia coli	GCA_028830865.1	DANMKK010000090.1	1381	2538	+
Escherichia coli	GCA_028830865.1	DANMKK010000137.1	1422	2579	+
Escherichia coli	GCA_028922145.1	DANNVK010000096.1	1098	2255	+
Escherichia coli	GCA_028922145.1	DANNVK010000144.1	1138	2295	+
Escherichia coli	GCA_029469365.1	DAODFY010000097.1	1097	2254	+
Escherichia coli	GCA_029469365.1	DAODFY010000180.1	1135	2292	+
Escherichia coli	GCA_029516395.1	DAOETM010000081.1	1008	2165	+
Escherichia coli	GCA_029516395.1	DAOETM010000145.1	1029	2186	+

Escherichia coli	GCA_029706505.1	DAOJAP010000142.1	1336	2493	+
Escherichia coli	GCA_029706505.1	DAOJAP010000193.1	1169	2326	+
Escherichia coli	GCA_031778115.1	DAPNUU010000168.1	1403	2560	+
Escherichia coli	GCA_031778115.1	DAPNUU010000235.1	1177	2334	+
Escherichia coli	GCA_032285135.1	DAPXYT010000087.1	1128	2285	+
Escherichia coli	GCA_032285135.1	DAPXYT010000166.1	402	1559	+
Escherichia coli	GCA_032285135.1	DAPXYT010000167.1	402	1559	+
Escherichia coli	GCA_032285205.1	DAPXYU010000077.1	1490	2647	+
Escherichia coli	GCA_032285205.1	DAPXYU010000130.1	1169	2326	+
Escherichia coli	GCA_032285685.1	DAPXZH010000037.1	1129	2286	+
Escherichia coli	GCA_032285685.1	DAPXZH010000079.1	1169	2326	+
Escherichia coli	GCA_032285685.1	DAPXZH010000080.1	1169	2326	+
Escherichia coli	GCA_032285765.1	DAPXZI010000061.1	1491	2648	+
Escherichia coli	GCA_032285765.1	DAPXZI010000107.1	1169	2326	+
Escherichia coli	GCA_032285705.1	DAPXZJ010000156.1	1096	2253	+
Escherichia coli	GCA_032285705.1	DAPXZJ010000262.1	1137	2294	+
Escherichia coli	GCA_032285745.1	DAPXZK010000119.1	1128	2285	+
Escherichia coli	GCA_032285745.1	DAPXZK010000205.1	1169	2326	+
Escherichia coli	GCA_032286305.1	DAPYAA010000118.1	1127	2284	+
Escherichia coli	GCA_032286305.1	DAPYAA010000201.1	1168	2325	+
Escherichia coli	GCA_032286945.1	DAPYBC010000099.1	1138	2295	+
Escherichia coli	GCA_032286945.1	DAPYBC010000170.1	1137	2294	+
Escherichia coli	GCA_032310475.1	DAPZBO010000135.1	892	2049	+
Escherichia coli	GCA_032310475.1	DAPZBO010000270.1	942	2099	+
Escherichia coli	GCA_032572315.1	DAQDDW010000064.1	1403	2560	+
Escherichia coli	GCA_032572315.1	DAQDDW010000121.1	1444	2601	+
Escherichia coli	GCA_032633685.1	DAQDXW010000042.1	1547	2704	+
Escherichia coli	GCA_032633685.1	DAQDXW010000090.1	1423	2580	+
Escherichia coli	GCA_032633665.1	DAQDXX010000035.1	1383	2540	+
Escherichia coli	GCA_032633665.1	DAQDXX010000066.1	1424	2581	+
Escherichia coli	GCA_033023035.1	DAQMUO010000076.1	1383	2540	+
Escherichia coli	GCA_033023035.1	DAQMUO010000127.1	1424	2581	+
Escherichia coli	GCA_033406835.1	DARBPF010000057.1	1714	2871	+
Escherichia coli	GCA_033406835.1	DARBPF010000151.1	1423	2580	+
Escherichia coli	GCA_033407035.1	DARBPJ010000092.1	1383	2540	+
Escherichia coli	GCA_033407035.1	DARBPJ010000161.1	1424	2581	+
Escherichia coli	GCA_033407275.1	DARBPY010000031.1	1714	2871	+
Escherichia coli	GCA_033407275.1	DARBPY010000066.1	1755	2912	+
Escherichia coli	GCA_019371395.1	JAEMZP010000064.1	1462	2616	+
Escherichia coli	GCA_019370195.1	JAEOBM010000043.1	1715	2869	+
Escherichia coli	GCA_019366595.1	JAEOHT010000098.1	1461	2615	+
Escherichia coli	GCA_019366165.1	JAEOIR010000073.1	4886	6040	-
Escherichia coli	GCA_019365285.1	JAEOKV010000074.1	7778	8932	+
Escherichia coli	GCA_028755825.1	JAIQMT010000115.1	1123	2277	-
Escherichia coli	GCA_028756175.1	JAIQNL010000152.1	1468	2622	+
Escherichia coli	GCA_023095725.1	JALLYW010000032.1	1207	2361	+
Escherichia coli	GCA_023095905.1	JALLZG010000090.1	1207	2361	+
Escherichia coli	GCA_023095915.1	JALLZH010000044.1	2374	3528	+
Escherichia coli	GCA_023095935.1	JALLZJ010000090.1	1207	2361	+
Escherichia coli	GCA_023097055.1	JALMAQ010000071.1	1172	2326	+
Escherichia coli	GCA_023097235.1	JALMBB010000114.1	301	1455	-
Salmonella enterica	GCA_030094115.1	JAMUIC010000030.1	224	1378	-
Escherichia coli	GCA_024754685.1	JANTIV010000066.1	1310	2464	-
Escherichia coli	GCA_024754805.1	JANTKT010000093.1	332	1486	-

Escherichia coli	GCA_024755625.1	JANTLF010000083.1	1144	2298	+
Escherichia coli	GCA_030712625.1	JAUOXB010000107.1	301	1455	-
Escherichia coli	GCA_030763085.1	JAUOYY010000053.1	1175	2329	+
Escherichia coli	GCA_032661265.1	JAVADQ010000071.1	1240	2394	+
Escherichia coli	GCA_032661065.1	JAVADU010000053.1	4619	5773	-
Escherichia coli	GCA_032661005.1	JAVADX010000105.1	335	1489	-
Escherichia coli	GCA_032660985.1	JAVADZ010000050.1	8419	9573	+

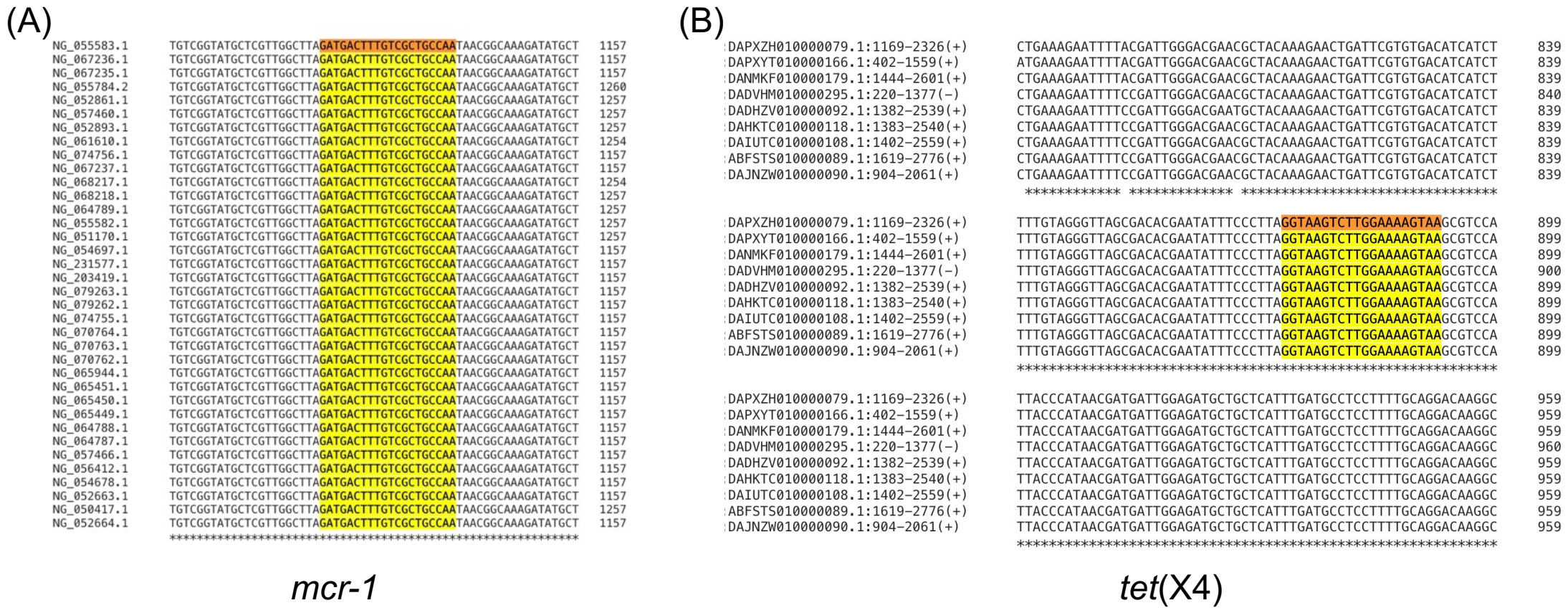

Fig. S1 Conservation analysis of the *mcr-1* and *tet*(X4) protospacer sequences. The 36 known *mcr-1* variants were analyzed (A). The 383 available *tet*(X4) sequences were downloaded from MicroBIGG-E database, and 9 variants were identified using cd-hit-est analysis (B). The protospacers (highlighted regions) were conserved among the sequences.

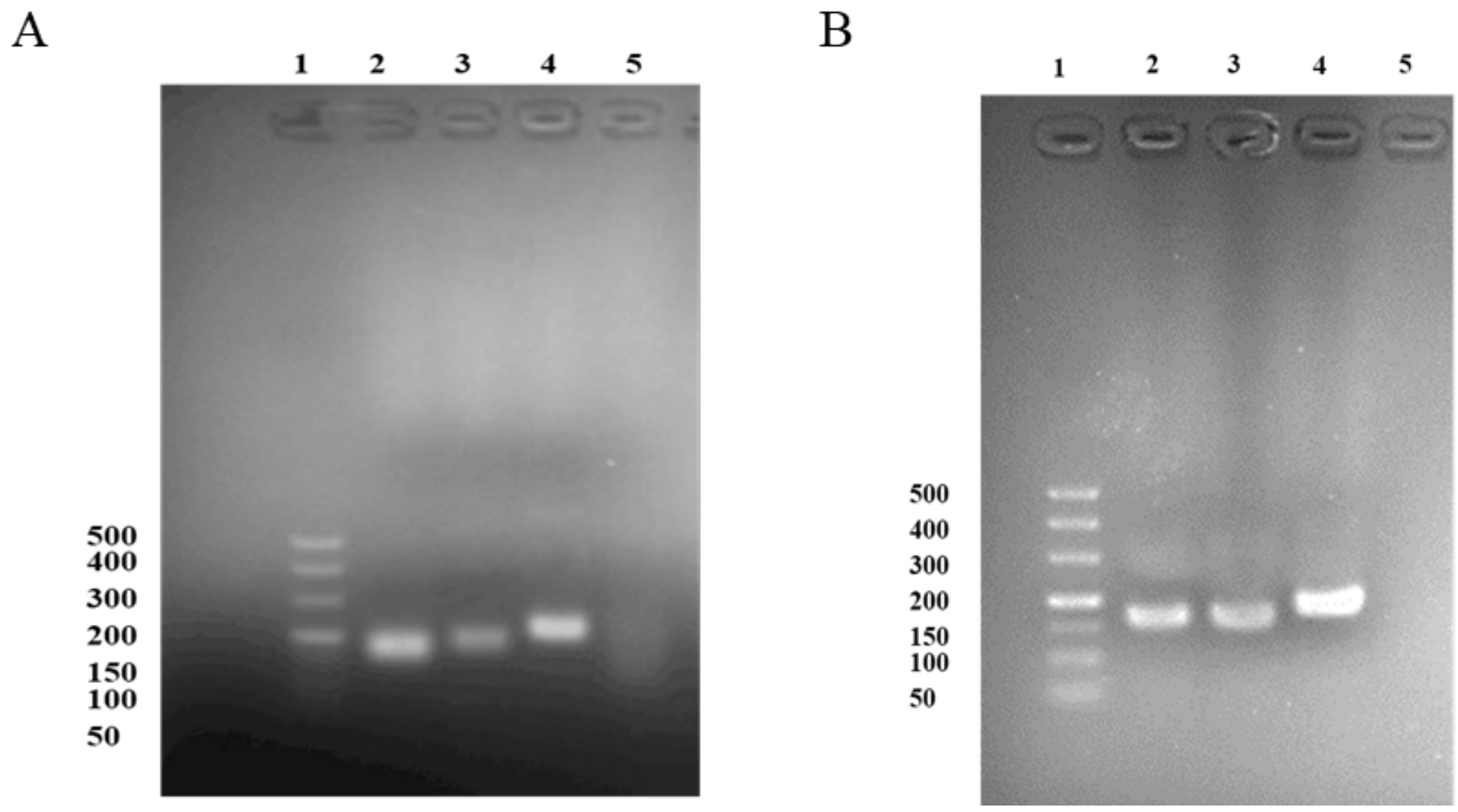

Fig. S2 Electrophoresis results of the *mcr-1* (A) and *tet*(X4) (B) RPA amplification products, respectively.

Note: 1: Marker 500; 2: The amplification products with the pair set 1; 3: The amplification products with the pair set 2; 4: The amplification products with the pair set 3; 5: Negative control.

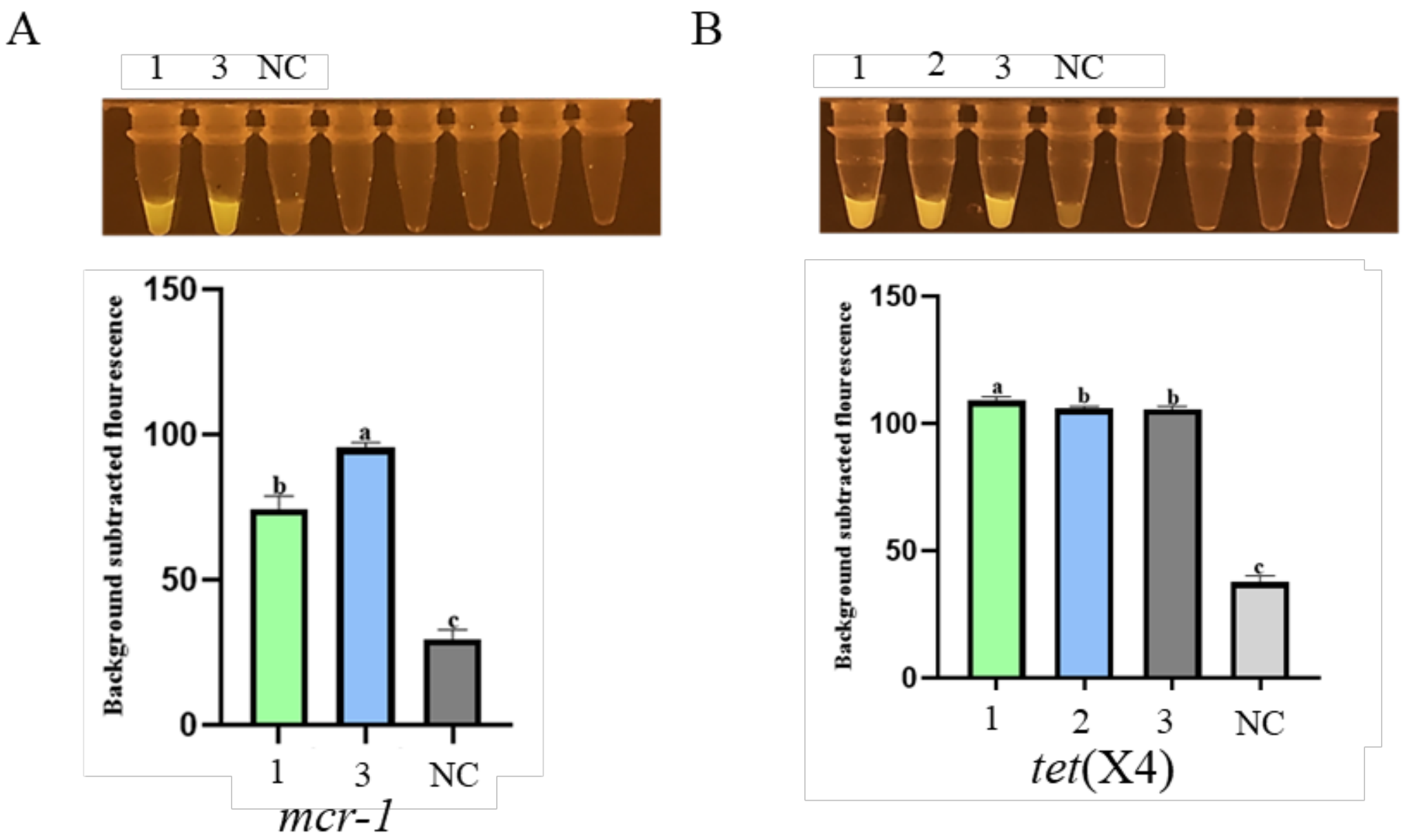

Fig. S3 Efficiency and fluorescence intensity of different RPA primer sets to activate the CRISPR-Cas12b detection systems. Different letters on the top of columns denote significant differences ($P < 0.05$, ANOVA). Error bars represent means ± SEM (n = 3 replicates).

Note: 1: The pair set 1; 2: The pair set 2; 3: The pair set 3; NC: Negative control.

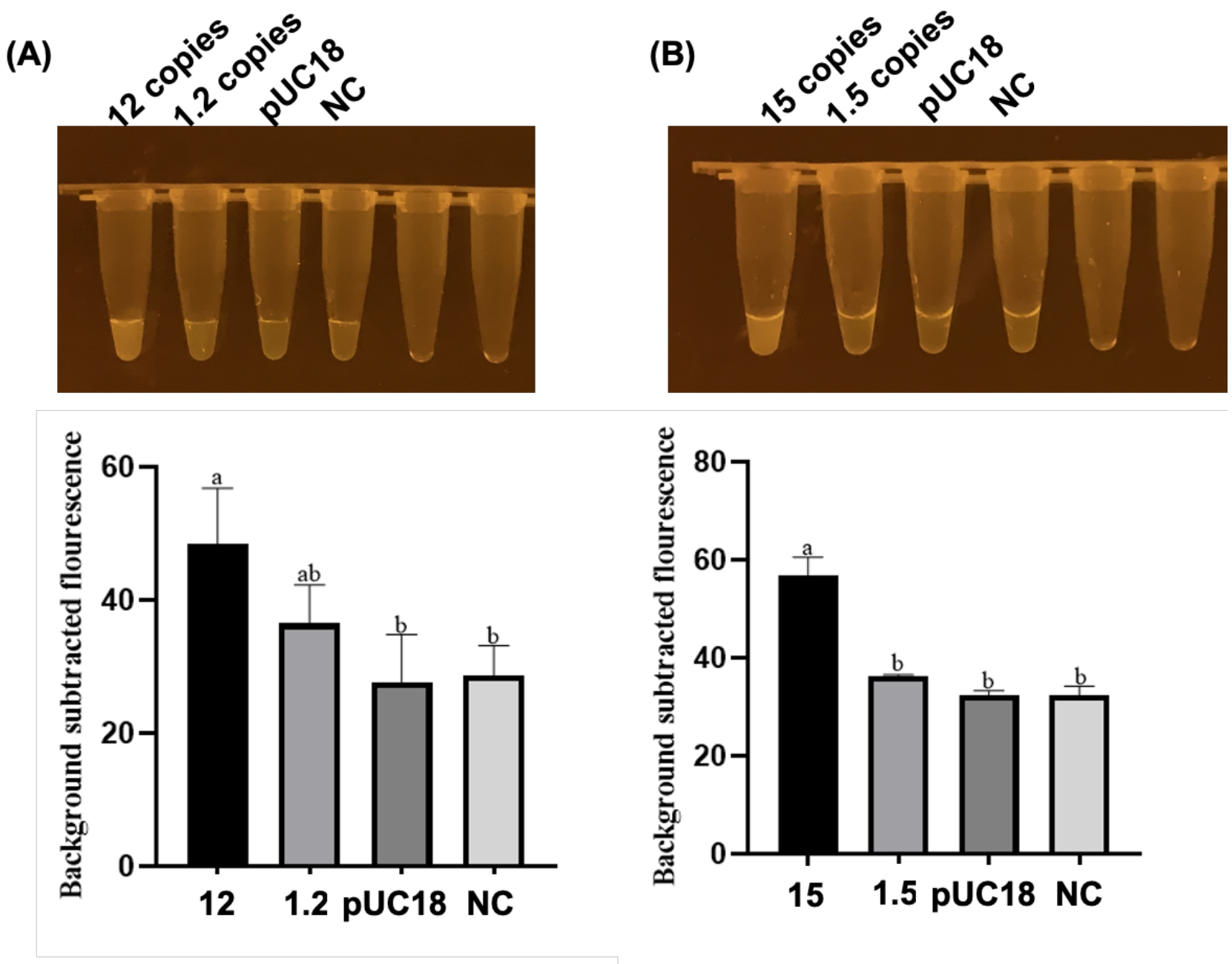

Fig. S4 the sensitivity assessment and fluorescence intensity of the one-tube RPA-CRISPR-Cas12b-based *mcr-1* (A) and *tet*(X4) (B) detection system. pUC18 plasmid (the vector used for *mcr-1* and *tet*(X4) dilution) was included in the assay. NC: negative control. Different letters on the top of columns denote significant differences ($P < 0.05$, ANOVA). Error bars represent means ± SEM (n = 3 replicates). The 'Background subtracted fluorescence' values were fluorescence values recorded in the reaction tubes minus the average florescence value obtained

from three blank tubes.

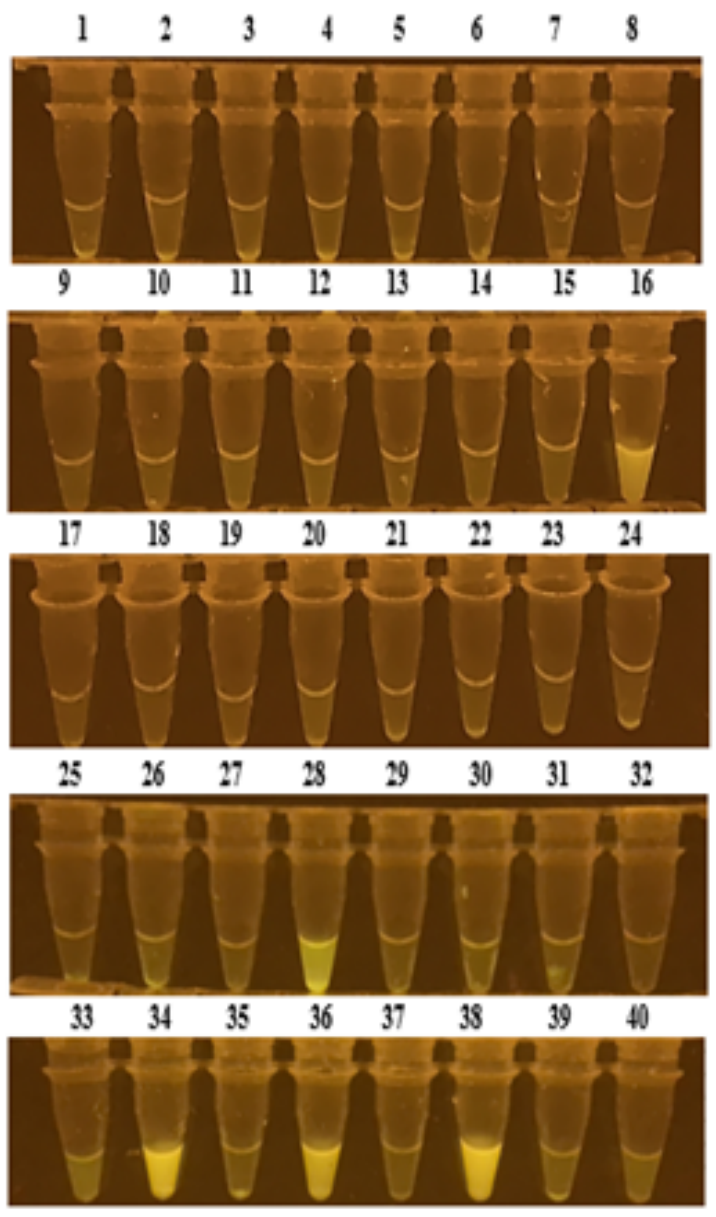

	1	2	3	4	5
CRISPR	-	-	-	-	-
Culture	-	-	-	-	-
	6	7	8	9	10
CRISPR	-	-	-	-	-
Culture	-	-	-	-	-
	11	12	13	14	15
CRISPR	-	-	-	-	-
Culture	-	-	-	-	-
	16	17	18	19	20
CRISPR	+	-	-	-	-
Culture	-	-	-	-	-
	21	22	23	24	25
CRISPR	-	-	-	-	-
Culture	-	-	-	-	-
	26	27	28	29	30
CRISPR	-	-	+	-	-
Culture	-	-	-	-	-
	31	32	33	34	35
CRISPR	-	-	+	+	-
Culture	-	-	-	+	-
	36	37	38	39	40
CRISPR	+	-	+	-	-
Culture	+	-	+	-	-

+:positive ,-:negative

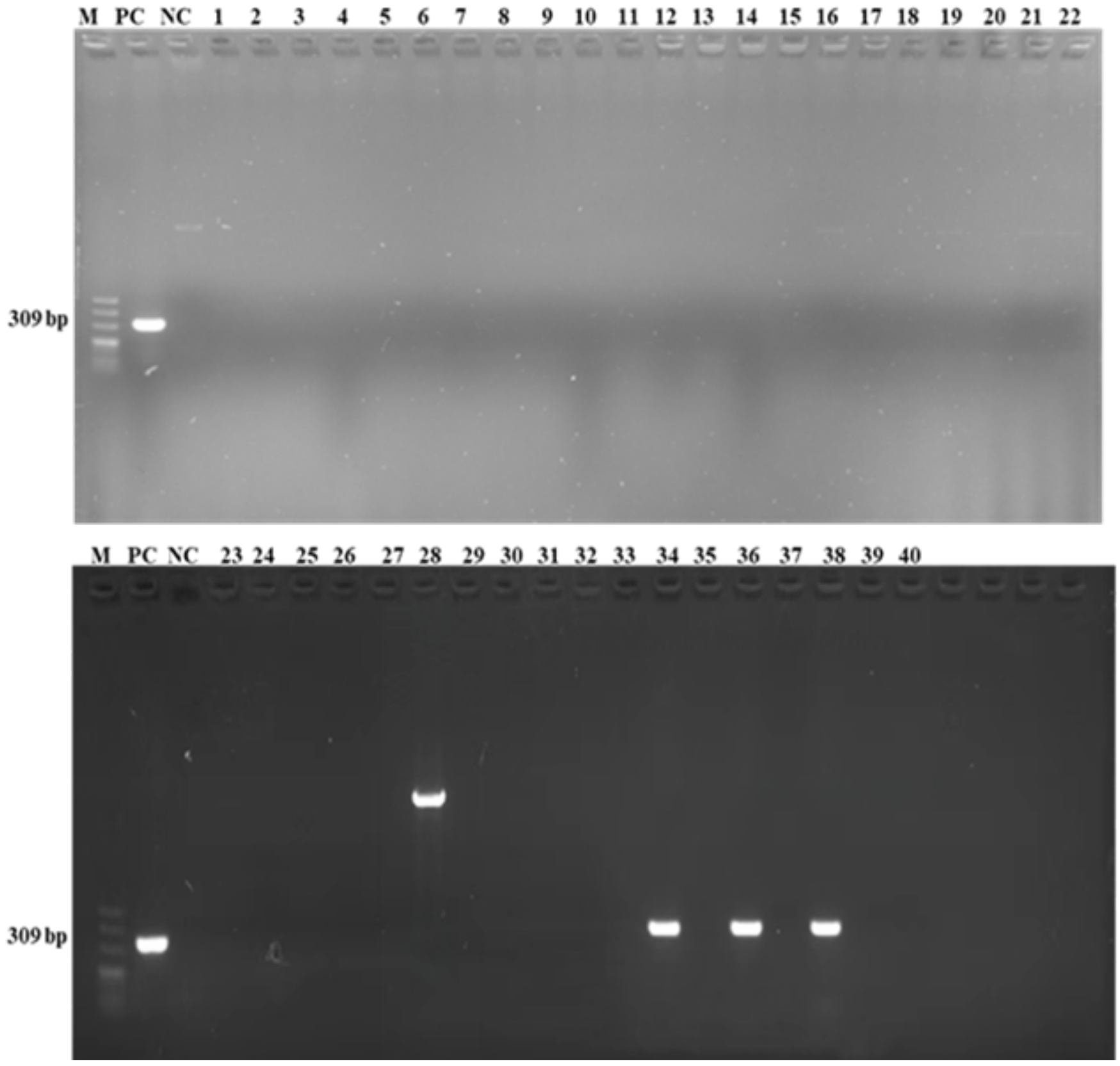

Fig. S5 Detection results of clinical samples by the RPA-CRISPR-Cas12b-based *mcr-1* detection system and the conventional cultivation-based method. Typical single colonies with inconsistent morphology (~20 colonies) were selected by colony PCR to determine the presence of *mcr-1*. The expected size was 309 bp for the *mcr-1* PCR products, and the band in sample 28 was a contamination.

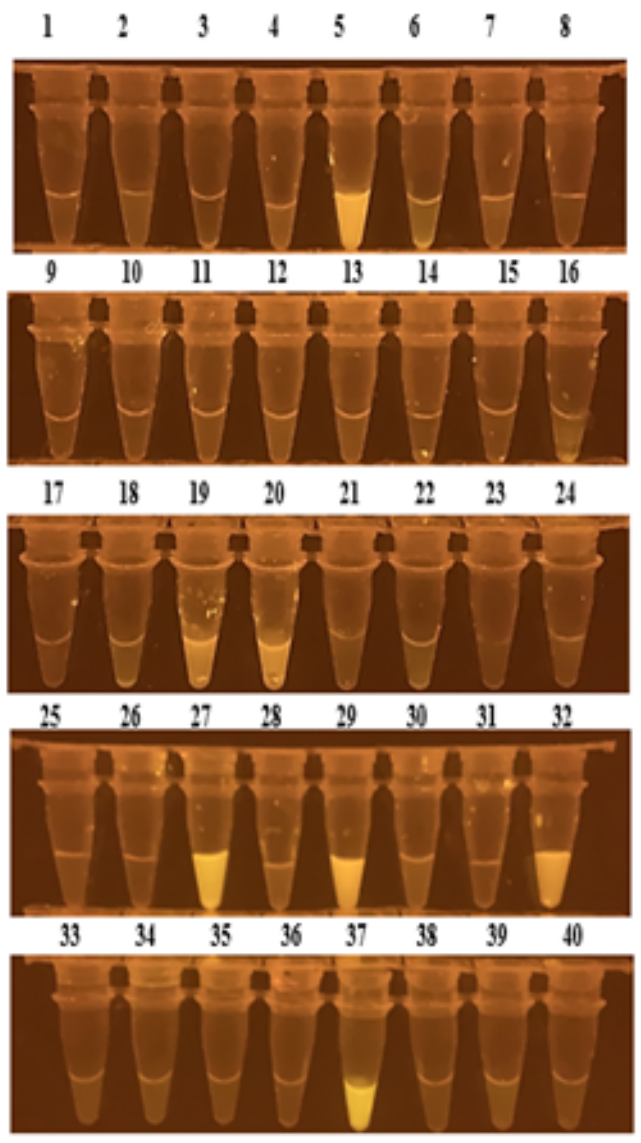

	1	2	3	4	5
CRISPR	-	-	-	-	+
Culture	-	-	-	-	+
	6	7	8	9	10
CRISPR	-	-	-	-	-
Culture	-	-	-	-	-
	11	12	13	14	15
CRISPR	-	-	-	-	-
Culture	-	-	-	-	-
	16	17	18	19	20
CRISPR	-	-	-	+	+
Culture	-	-	-	+	+
	21	22	23	24	25
CRISPR	-	-	-	-	-
Culture	-	-	-	-	-
	26	27	28	29	30
CRISPR	-	+	-	+	-
Culture	-	+	-	+	-
	31	32	33	34	35
CRISPR	-	+	-	-	-
Culture	-	+	-	-	-
	36	37	38	39	40
CRISPR	-	+	-	-	-
Culture	-	+	-	-	-

+:positive ,-:negative

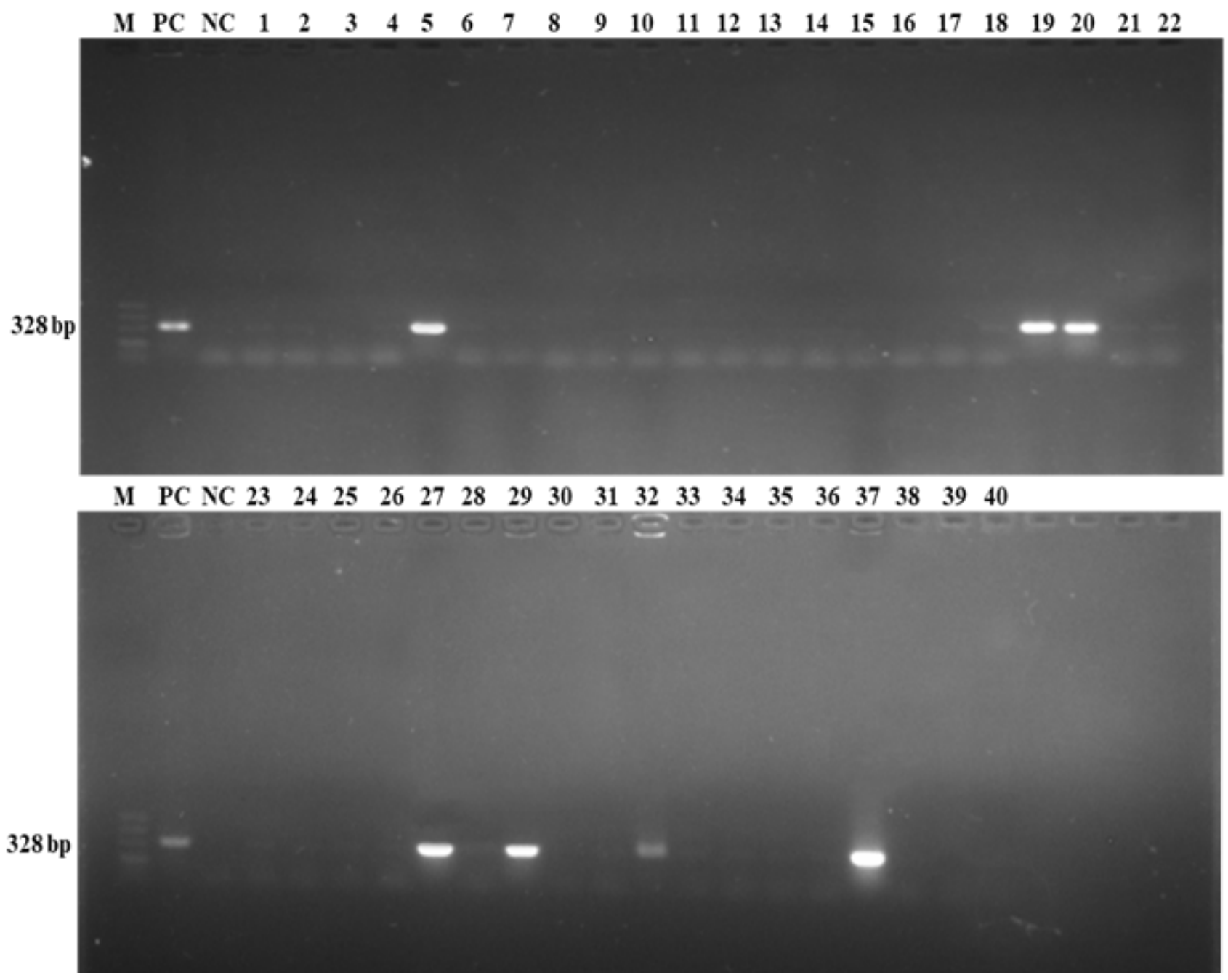

Fig. S6 Detection results of clinical samples by the RPA-CRISPR-Cas12b-based *tet*(X4) detection system and the conventional cultivation-based method. Typical single colonies with inconsistent morphology (~20 colonies) were selected by colony PCR to determine the presence of *tet*(X4).
