# Supplementary material for: Development and evaluation of rapid and accurate one-tube RPA-CRISPR-Cas12b-based detection of mcr-1 and tet(X4)
Source: Appl Microbiol Biotechnol. 2024 May 27;108(1):345. doi: 10.1007/s00253-024-13191-6 (PMC11129972; doi:10.1007/s00253-024-13191-6)
Supplement: Supplementary file 1 — Supplementary file1 (PDF 3017 KB) [file 253_2024_13191_MOESM1_ESM.pdf]

Applied Microbiology and Biotechnology

# Development and evaluation of rapid and accurate one-tube RPA-CRISPR-Cas12b-based detection of *mcr-1* and *tet*(X4)

**Yu Wang[1,2,3,4], Huan Chen[1,2,3,4], Qingyun Pan[1,2,3,4], Jing Wang[1,2,3,4], Xin'an Jiao[1,2,3,4*], Yunzeng Zhang[1,2,3,4*]**

[1] Jiangsu Co-Innovation Center for Prevention and Control of Important Animal Infectious Diseases and Zoonoses, Yangzhou University, Yangzhou 225009, China

[2] Jiangsu Key Laboratory of Zoonosis, Yangzhou University, Yangzhou 225009, China

[3] Joint International Research Laboratory of Agriculture and Agri-product Safety of the Ministry of Education, Yangzhou University, Yangzhou 225009, China

[4] Key Laboratory of Prevention and Control of Biological Hazard Factors (Animal Origin) for Agrifood Safety and Quality, Ministry of Agriculture of China, Yangzhou University, Yangzhou 225009, China

*** Correspondence:**

Yunzeng Zhang, yzzhang@yzu.edu.cn; Tel: +86-5145-87971136

Address: No. 48 Wenhui East Road, College of Bioscience and Biotechnology, Yangzhou University, Yangzhou, Jiangsu, China, 225009

Xin'an Jiao, jiao@yzu.edu.cn; Tel: +86-5145-87971136

Address: No. 48 Wenhui East Road, College of Bioscience and Biotechnology, Yangzhou University, Yangzhou, Jiangsu, China, 225009

Table S1. The accession numbers of the *mcr-1* sequences used for protospacer discovery.

| |
|---|
| NG_050417.1 |
| NG_051170.1 |
| NG_052861.1 |
| NG_052664.1 |
| NG_052663.1 |
| NG_052893.1 |
| NG_054678.1 |
| NG_054697.1 |
| NG_055582.1 |
| NG_055583.1 |
| NG_055784.2 |
| NG_056412.1 |
| NG_057466.1 |
| NG_057460.1 |
| NG_061610.1 |
| NG_064787.1 |
| NG_064788.1 |
| NG_064789.1 |
| NG_065449.1 |
| NG_065450.1 |
| NG_065451.1 |
| NG_065944.1 |
| NG_067235.1 |
| NG_067236.1 |
| NG_067237.1 |
| NG_068217.1 |
| NG_068218.1 |
| NG_070762.1 |
| NG_070763.1 |
| NG_070764.1 |
| NG_074755.1 |
| NG_074756.1 |
| NG_079262.1 |
| NG_079263.1 |
| NG_203419.1 |
| NG_231577.1 |

Table S2. The accession numbers of the *tet* (X4) sequences used for protospacer discovery.

| Scientific name | Assembly | Contig | Start | Stop | Strand |
|---|---|---|---|---|---|
| *Salmonella enterica* subsp. *enterica* serovar Agona | GCA_004183755.1 | AAABRY010000064.1 | 1116 | 2273 | + |
| *Salmonella enterica* subsp. *enterica* serovar Agona | GCA_004183755.1 | AAABRY010000091.1 | 1147 | 2304 | + |
| *Salmonella enterica* subsp. *enterica* serovar Stanley | GCA_007249975.1 | AAHLAN010000051.1 | 1006 | 2163 | + |
| *Salmonella enterica* subsp. *enterica* serovar Livingstone | GCA_007264485.1 | AAHMBT010000084.1 | 1064 | 2221 | + |
| *Salmonella enterica* subsp. *enterica* serovar Livingstone | GCA_007264485.1 | AAHMBT010000116.1 | 1095 | 2252 | + |
| *Salmonella enterica* | GCA_008167565.1 | AAJTXD010000043.1 | 1382 | 2539 | + |
| *Salmonella enterica* | GCA_008167565.1 | AAJTXD010000071.1 | 1383 | 2540 | + |
| *Salmonella enterica* subsp. *enterica* serovar Typhimurium | GCA_008201095.1 | AAJWIR010000186.1 | 1093 | 2250 | + |
| *Salmonella enterica* subsp. *enterica* serovar Typhimurium | GCA_008201095.1 | AAJWIR010000250.1 | 1124 | 2281 | + |
| *Salmonella enterica* subsp. *enterica* serovar Typhimurium | GCA_008540555.1 | AAKSZD010000064.1 | 1108 | 2265 | + |
| *Salmonella enterica* subsp. *enterica* serovar Typhimurium | GCA_008540555.1 | AAKSZD010000121.1 | 1135 | 2292 | + |
| *Salmonella enterica* subsp. *enterica* serovar Agona | GCA_008576375.1 | AAKUGF010000071.1 | 1383 | 2540 | + |
| *Salmonella enterica* subsp. *enterica* serovar Agona | GCA_008576375.1 | AAKUGF010000103.1 | 1062 | 2219 | + |
| *Salmonella enterica* subsp. *enterica* serovar Kentucky | GCA_010931195.1 | AAPAUS010000037.1 | 1619 | 2776 | + |
| *Salmonella enterica* subsp. *enterica* serovar Kentucky | GCA_010931195.1 | AAPAUS010000054.1 | 1384 | 2541 | + |
| *Salmonella enterica* subsp. *enterica* serovar Stanley | GCA_011326515.1 | AAPUUC010000047.1 | 1101 | 2258 | + |
| *Salmonella enterica* subsp. *enterica* serovar Stanley | GCA_011326515.1 | AAPUUC010000091.1 | 1132 | 2289 | + |
| *Shigella sonnei* | GCA_013458775.1 | AATWGR010000223.1 | 1102 | 2259 | + |
| *Shigella sonnei* | GCA_013458775.1 | AATWGR010000452.1 | 889 | 2046 | + |
| *Salmonella enterica* | GCA_017796145.1 | AAZUQE010000053.1 | 1042 | 2199 | + |
| *Salmonella enterica* | GCA_017796145.1 | AAZUQE010000079.1 | 1073 | 2230 | + |
| *Salmonella enterica* | GCA_017802095.1 | AAZVAB010000035.1 | 1107 | 2264 | + |
| *Salmonella enterica* | GCA_017802095.1 | AAZVAB010000064.1 | 1136 | 2293 | + |
| *Salmonella enterica* | GCA_017834535.1 | AAZWSE010000064.1 | 1066 | 2223 | + |
| *Salmonella enterica* | GCA_017834535.1 | AAZWSE010000096.1 | 1097 | 2254 | + |
| *Salmonella enterica* | GCA_017865385.1 | AAZXRY010000094.1 | 1100 | 2257 | + |
| *Salmonella enterica* | GCA_017865385.1 | AAZXRY010000137.1 | 1133 | 2290 | + |
| *Salmonella enterica* subsp. *enterica* serovar Kentucky | GCA_018978845.1 | ABAYCX010000037.1 | 1619 | 2776 | + |
| *Salmonella enterica* subsp. *enterica* serovar Kentucky | GCA_018978845.1 | ABAYCX010000053.1 | 1424 | 2581 | + |
| *Escherichia coli* | GCA_022421445.1 | ABFGCA010000106.1 | 1063 | 2220 | + |
| *Escherichia coli* | GCA_022421445.1 | ABFGCA010000156.1 | 1094 | 2251 | + |
| *Escherichia coli* | GCA_022437005.1 | ABFGOC010000102.1 | 1123 | 2280 | + |
| *Escherichia coli* | GCA_022437005.1 | ABFGOC010000162.1 | 345 | 1502 | + |
| *Escherichia coli* | GCA_022633235.1 | ABFLGQ010000206.1 | 1113 | 2270 | + |
| *Escherichia coli* | GCA_022633235.1 | ABFLGQ010000343.1 | 1089 | 2246 | + |
| *Escherichia coli* | GCA_022636115.1 | ABFLMC010000133.1 | 1069 | 2226 | + |
| *Escherichia coli* | GCA_022636115.1 | ABFLMC010000181.1 | 905 | 2062 | + |
| *Escherichia coli* | GCA_022947075.1 | ABFSTS010000089.1 | 1619 | 2776 | + |
| *Escherichia coli* | GCA_022947075.1 | ABFSTS010000123.1 | 1424 | 2581 | + |
| *Escherichia coli* | GCA_023310495.1 | ABGCRY010000102.1 | 1383 | 2540 | + |
| *Escherichia coli* | GCA_023310495.1 | ABGCRY010000157.1 | 1424 | 2581 | + |
| *Escherichia coli* | GCA_023310715.1 | ABGCSG010000094.1 | 1619 | 2776 | + |
| *Escherichia coli* | GCA_023310715.1 | ABGCSG010000142.1 | 1424 | 2581 | + |
| *Escherichia coli* | GCA_023403425.1 | ABGFKC010000078.1 | 1304 | 2461 | + |
| *Escherichia coli* | GCA_023403425.1 | ABGFKC010000149.1 | 1345 | 2502 | + |
| *Escherichia coli* | GCA_025027415.1 | ABIBFN010000144.1 | 1383 | 2540 | + |
| *Escherichia coli* | GCA_025027415.1 | ABIBFN010000271.1 | 1424 | 2581 | + |
| *Salmonella enterica* | GCA_026040715.1 | ABJEAM010000037.1 | 1946 | 3103 | + |
| *Salmonella enterica* | GCA_026040715.1 | ABJEAM010000056.1 | 1424 | 2581 | + |
| *Salmonella enterica* | GCA_031661075.1 | ABMPIU010000019.1 | 1619 | 2776 | + |
| *Salmonella enterica* | GCA_031661075.1 | ABMPIU010000035.1 | 1424 | 2581 | + |
| *Escherichia coli* | GCA_918364955.1 | CAKKEC010000080.1 | 7094 | 8248 | - |

| | | | | | |
|---|---|---|---|---|---|
| *Escherichia coli* | GCA_021398675.1 | CP090284.1 | 53354 | 54508 | + |
| *Escherichia coli* | GCA_023099705.2 | CP101799.1 | 34654 | 35808 | - |
| *Escherichia coli* | GCA_023098635.2 | CP101801.1 | 53354 | 54508 | + |
| *Escherichia coli* | GCA_012297045.1 | DAAZEY010000048.1 | 1383 | 2540 | + |
| *Escherichia coli* | GCA_012297045.1 | DAAZEY010000090.1 | 897 | 2054 | + |
| *Escherichia coli* | GCA_012502375.1 | DABAJL010000078.1 | 1383 | 2540 | + |
| *Escherichia coli* | GCA_012502375.1 | DABAJL010000115.1 | 1384 | 2541 | + |
| *Escherichia coli* | GCA_012501995.1 | DABAJO010000069.1 | 1403 | 2560 | + |
| *Escherichia coli* | GCA_012501995.1 | DABAJO010000097.1 | 1404 | 2561 | + |
| *Escherichia coli* | GCA_013077765.1 | DABIHN010000105.1 | 1637 | 2794 | + |
| *Escherichia coli* | GCA_013077765.1 | DABIHN010000171.1 | 1404 | 2561 | + |
| *Escherichia coli* | GCA_013077305.1 | DABIID010000063.1 | 1403 | 2560 | + |
| *Escherichia coli* | GCA_013077305.1 | DABIID010000102.1 | 1404 | 2561 | + |
| *Escherichia coli* | GCA_013077385.1 | DABIIH010000081.1 | 1383 | 2540 | + |
| *Escherichia coli* | GCA_013077385.1 | DABIIH010000119.1 | 1384 | 2541 | + |
| *Escherichia coli* | GCA_013077405.1 | DABIII010000081.1 | 1383 | 2540 | + |
| *Escherichia coli* | GCA_013077405.1 | DABIII010000118.1 | 1384 | 2541 | + |
| *Escherichia coli* | GCA_014657275.1 | DABTSW010000162.1 | 1044 | 2201 | + |
| *Escherichia coli* | GCA_014657275.1 | DABTSW010000311.1 | 1143 | 2300 | + |
| *Escherichia coli* | GCA_017044295.1 | DADHZV010000092.1 | 1382 | 2539 | + |
| *Escherichia coli* | GCA_017044295.1 | DADHZV010000194.1 | 1423 | 2580 | + |
| *Escherichia coli* | GCA_017044335.1 | DADHZW010000055.1 | 1382 | 2539 | + |
| *Escherichia coli* | GCA_017044335.1 | DADHZW010000101.1 | 1423 | 2580 | + |
| *Escherichia coli* | GCA_017044315.1 | DADHZX010000055.1 | 1382 | 2539 | + |
| *Escherichia coli* | GCA_017044315.1 | DADHZX010000108.1 | 1423 | 2580 | + |
| *Escherichia coli* | GCA_017044255.1 | DADHZZ010000066.1 | 1382 | 2539 | + |
| *Escherichia coli* | GCA_017044255.1 | DADHZZ010000102.1 | 1423 | 2580 | + |
| *Escherichia coli* | GCA_017044615.1 | DADIAC010000054.1 | 1382 | 2539 | + |
| *Escherichia coli* | GCA_017044615.1 | DADIAC010000108.1 | 1423 | 2580 | + |
| *Escherichia coli* | GCA_017044575.1 | DADIAD010000107.1 | 1382 | 2539 | + |
| *Escherichia coli* | GCA_017044575.1 | DADIAD010000195.1 | 1423 | 2580 | + |
| *Escherichia coli* | GCA_017044475.1 | DADIAF010000058.1 | 1382 | 2539 | + |
| *Escherichia coli* | GCA_017044475.1 | DADIAF010000098.1 | 1423 | 2580 | + |
| *Escherichia coli* | GCA_017044535.1 | DADIAH010000069.1 | 1382 | 2539 | + |
| *Escherichia coli* | GCA_017044535.1 | DADIAH010000132.1 | 1423 | 2580 | + |
| *Escherichia coli* | GCA_017044895.1 | DADIAS010000040.1 | 1382 | 2539 | + |
| *Escherichia coli* | GCA_017044895.1 | DADIAS010000076.1 | 1423 | 2580 | + |
| *Escherichia coli* | GCA_017045015.1 | DADIAT010000100.1 | 1382 | 2539 | + |
| *Escherichia coli* | GCA_017045015.1 | DADIAT010000176.1 | 1423 | 2580 | + |
| *Escherichia coli* | GCA_017044875.1 | DADIAW010000069.1 | 1382 | 2539 | + |
| *Escherichia coli* | GCA_017044875.1 | DADIAW010000129.1 | 1423 | 2580 | + |
| *Escherichia coli* | GCA_017045215.1 | DADIBC010000067.1 | 1382 | 2539 | + |
| *Escherichia coli* | GCA_017045215.1 | DADIBC010000134.1 | 1423 | 2580 | + |
| *Escherichia coli* | GCA_017045435.1 | DADIBL010000062.1 | 1382 | 2539 | + |
| *Escherichia coli* | GCA_017045435.1 | DADIBL010000107.1 | 1423 | 2580 | + |
| *Escherichia coli* | GCA_017045275.1 | DADIBR010000060.1 | 1382 | 2539 | + |
| *Escherichia coli* | GCA_017045275.1 | DADIBR010000116.1 | 1423 | 2580 | + |
| *Escherichia coli* | GCA_017045795.1 | DADICO010000040.1 | 1570 | 2727 | + |
| *Escherichia coli* | GCA_017045795.1 | DADICO010000077.1 | 1423 | 2580 | + |
| *Escherichia coli* | GCA_017046315.1 | DADIDJ010000078.1 | 1382 | 2539 | + |
| *Escherichia coli* | GCA_017046315.1 | DADIDJ010000172.1 | 1423 | 2580 | + |
| *Escherichia coli* | GCA_017046395.1 | DADIDK010000104.1 | 1382 | 2539 | + |
| *Escherichia coli* | GCA_017046395.1 | DADIDK010000177.1 | 1423 | 2580 | + |
| *Escherichia coli* | GCA_017046635.1 | DADIEF010000033.1 | 1382 | 2539 | + |

| | | | | | |
|---|---|---|---|---|---|
| *Escherichia coli* | GCA_017046635.1 | DADIEF010000078.1 | 1423 | 2580 | + |
| *Escherichia coli* | GCA_017046555.1 | DADIEH010000094.1 | 1382 | 2539 | + |
| *Escherichia coli* | GCA_017046555.1 | DADIEH010000171.1 | 1423 | 2580 | + |
| *Escherichia coli* | GCA_017046695.1 | DADIEK010000097.1 | 1382 | 2539 | + |
| *Escherichia coli* | GCA_017046695.1 | DADIEK010000219.1 | 1423 | 2580 | + |
| *Escherichia coli* | GCA_017046955.1 | DADIEP010000050.1 | 1382 | 2539 | + |
| *Escherichia coli* | GCA_017046955.1 | DADIEP010000093.1 | 1423 | 2580 | + |
| *Escherichia coli* | GCA_017047015.1 | DADIEY010000089.1 | 1382 | 2539 | + |
| *Escherichia coli* | GCA_017047015.1 | DADIEY010000152.1 | 1423 | 2580 | + |
| *Escherichia coli* | GCA_017046895.1 | DADIFD010000068.1 | 1382 | 2539 | + |
| *Escherichia coli* | GCA_017046895.1 | DADIFD010000112.1 | 1423 | 2580 | + |
| *Escherichia coli* | GCA_017047535.1 | DADIFO010000058.1 | 1382 | 2539 | + |
| *Escherichia coli* | GCA_017047535.1 | DADIFO010000109.1 | 1423 | 2580 | + |
| *Escherichia coli* | GCA_017047455.1 | DADIFR010000124.1 | 1382 | 2539 | + |
| *Escherichia coli* | GCA_017047455.1 | DADIFR010000254.1 | 1423 | 2580 | + |
| *Escherichia coli* | GCA_017910375.1 | DADVHM010000295.1 | 220 | 1377 | - |
| *Escherichia coli* | GCA_017910375.1 | DADVHM010000442.1 | 889 | 2046 | + |
| *Escherichia coli* | GCA_018022355.1 | DADWPG010000048.1 | 1382 | 2539 | + |
| *Escherichia coli* | GCA_018022355.1 | DADWPG010000094.1 | 1423 | 2580 | + |
| *Escherichia coli* | GCA_018295365.1 | DAEAWD010000085.1 | 1384 | 2541 | + |
| *Escherichia coli* | GCA_018295365.1 | DAEAWD010000128.1 | 906 | 2063 | + |
| *Escherichia coli* | GCA_018295395.1 | DAEAWE010000104.1 | 1384 | 2541 | + |
| *Escherichia coli* | GCA_018295395.1 | DAEAWE010000161.1 | 936 | 2093 | + |
| *Escherichia coli* | GCA_018295325.1 | DAEAWF010000046.1 | 1195 | 2352 | + |
| *Escherichia coli* | GCA_018295325.1 | DAEAWF010000089.1 | 1168 | 2325 | + |
| *Escherichia coli* | GCA_018295385.1 | DAEAWH010000104.1 | 1403 | 2560 | + |
| *Escherichia coli* | GCA_018295385.1 | DAEAWH010000153.1 | 1444 | 2601 | + |
| *Escherichia coli* | GCA_018295425.1 | DAEAWK010000102.1 | 1129 | 2286 | + |
| *Escherichia coli* | GCA_018295425.1 | DAEAWK010000177.1 | 1170 | 2327 | + |
| *Escherichia coli* | GCA_018295465.1 | DAEAWN010000053.1 | 1384 | 2541 | + |
| *Escherichia coli* | GCA_018295465.1 | DAEAWN010000079.1 | 1425 | 2582 | + |
| *Escherichia coli* | GCA_018295445.1 | DAEAWO010000093.1 | 1096 | 2253 | + |
| *Escherichia coli* | GCA_018295445.1 | DAEAWO010000147.1 | 1136 | 2293 | + |
| *Escherichia coli* | GCA_018295525.1 | DAEAWR010000104.1 | 1383 | 2540 | + |
| *Escherichia coli* | GCA_018295525.1 | DAEAWR010000153.1 | 1424 | 2581 | + |
| *Escherichia coli* | GCA_018295535.1 | DAEAWS010000121.1 | 1496 | 2653 | + |
| *Escherichia coli* | GCA_018295535.1 | DAEAWS010000200.1 | 1132 | 2289 | + |
| *Escherichia coli* | GCA_018295565.1 | DAEAWU010000038.1 | 1129 | 2286 | + |
| *Escherichia coli* | GCA_018295565.1 | DAEAWU010000103.1 | 1169 | 2326 | + |
| *Escherichia coli* | GCA_018295645.1 | DAEAWV010000134.1 | 1129 | 2286 | + |
| *Escherichia coli* | GCA_018295645.1 | DAEAWV010000201.1 | 1170 | 2327 | + |
| *Escherichia coli* | GCA_018295585.1 | DAEAWW010000081.1 | 1129 | 2286 | + |
| *Escherichia coli* | GCA_018295585.1 | DAEAWW010000118.1 | 1169 | 2326 | + |
| *Escherichia coli* | GCA_018295865.1 | DAEAWX010000072.1 | 1129 | 2286 | + |
| *Escherichia coli* | GCA_018295865.1 | DAEAWX010000139.1 | 936 | 2093 | + |
| *Escherichia coli* | GCA_018295805.1 | DAEAWY010000096.1 | 1094 | 2251 | + |
| *Escherichia coli* | GCA_018295805.1 | DAEAWY010000139.1 | 1135 | 2292 | + |
| *Escherichia coli* | GCA_018295505.1 | DAEAWZ010000080.1 | 1403 | 2560 | + |
| *Escherichia coli* | GCA_018295505.1 | DAEAWZ010000133.1 | 1444 | 2601 | + |
| *Escherichia coli* | GCA_018295825.1 | DAEAXA010000115.1 | 1095 | 2252 | + |
| *Escherichia coli* | GCA_018295825.1 | DAEAXA010000173.1 | 936 | 2093 | + |
| *Escherichia coli* | GCA_018295665.1 | DAEAXB010000073.1 | 1490 | 2647 | + |
| *Escherichia coli* | GCA_018295665.1 | DAEAXB010000141.1 | 1169 | 2326 | + |
| *Escherichia coli* | GCA_018295685.1 | DAEAXC010000051.1 | 1128 | 2285 | + |

| | | | | | |
|---|---|---|---|---|---|
| *Escherichia coli* | GCA_018295685.1 | DAEAXC010000099.1 | 1169 | 2326 | + |
| *Escherichia coli* | GCA_018295765.1 | DAEAXD010000070.1 | 1383 | 2540 | + |
| *Escherichia coli* | GCA_018295765.1 | DAEAXD010000127.1 | 1424 | 2581 | + |
| *Escherichia coli* | GCA_018295605.1 | DAEAXE010000052.1 | 1384 | 2541 | + |
| *Escherichia coli* | GCA_018295605.1 | DAEAXE010000144.1 | 906 | 2063 | + |
| *Escherichia coli* | GCA_018295705.1 | DAEAXF010000096.1 | 1096 | 2253 | + |
| *Escherichia coli* | GCA_018295705.1 | DAEAXF010000154.1 | 1136 | 2293 | + |
| *Escherichia coli* | GCA_018295745.1 | DAEAXG010000102.1 | 1383 | 2540 | + |
| *Escherichia coli* | GCA_018295745.1 | DAEAXG010000154.1 | 1424 | 2581 | + |
| *Escherichia coli* | GCA_018295785.1 | DAEAXH010000097.1 | 1094 | 2251 | + |
| *Escherichia coli* | GCA_018295785.1 | DAEAXH010000142.1 | 1135 | 2292 | + |
| *Escherichia coli* | GCA_018295725.1 | DAEAXI010000075.1 | 1128 | 2285 | + |
| *Escherichia coli* | GCA_018295725.1 | DAEAXI010000113.1 | 1169 | 2326 | + |
| *Escherichia coli* | GCA_018295625.1 | DAEAXJ010000097.1 | 1095 | 2252 | + |
| *Escherichia coli* | GCA_018295625.1 | DAEAXJ010000149.1 | 1136 | 2293 | + |
| *Escherichia coli* | GCA_021088305.1 | DAFGWP010000098.1 | 1383 | 2540 | + |
| *Escherichia coli* | GCA_021088305.1 | DAFGWP010000181.1 | 1424 | 2581 | + |
| *Escherichia coli* | GCA_021453045.1 | DAFLOI010000158.1 | 1379 | 2536 | + |
| *Escherichia coli* | GCA_021453045.1 | DAFLOI010000247.1 | 1420 | 2577 | + |
| *Escherichia coli* | GCA_021454425.1 | DAFLQZ010000159.1 | 1379 | 2536 | + |
| *Escherichia coli* | GCA_021454425.1 | DAFLQZ010000248.1 | 1420 | 2577 | + |
| *Klebsiella pneumoniae* | GCA_021933115.1 | DAFXYV010000074.1 | 1075 | 2232 | + |
| *Klebsiella pneumoniae* | GCA_021933115.1 | DAFXYV010000169.1 | 1114 | 2271 | + |
| *Klebsiella pneumoniae* | GCA_022026825.1 | DAGCMU010000057.1 | 1383 | 2540 | + |
| *Klebsiella pneumoniae* | GCA_022026825.1 | DAGCMU010000107.1 | 1424 | 2581 | + |
| *Klebsiella pneumoniae* | GCA_022026775.1 | DAGCMV010000058.1 | 1382 | 2539 | + |
| *Klebsiella pneumoniae* | GCA_022026775.1 | DAGCMV010000108.1 | 1423 | 2580 | + |
| *Klebsiella pneumoniae* | GCA_022355205.1 | DAGTSU010000032.1 | 1619 | 2776 | + |
| *Klebsiella pneumoniae* | GCA_022355205.1 | DAGTSU010000057.1 | 1384 | 2541 | + |
| *Escherichia coli* | GCA_023465755.1 | DAHEXP010000104.1 | 1040 | 2197 | + |
| *Escherichia coli* | GCA_023465755.1 | DAHEXP010000157.1 | 26 | 1183 | + |
| *Escherichia coli* | GCA_023674145.1 | DAHJKX010000211.1 | 220 | 1377 | - |
| *Escherichia coli* | GCA_023674145.1 | DAHJKX010000369.1 | 889 | 2046 | + |
| *Escherichia coli* | GCA_023778665.1 | DAHKSM010000062.1 | 1633 | 2790 | + |
| *Escherichia coli* | GCA_023778665.1 | DAHKSM010000103.1 | 1674 | 2831 | + |
| *Escherichia coli* | GCA_023779205.1 | DAHKTC010000118.1 | 1383 | 2540 | + |
| *Escherichia coli* | GCA_023779205.1 | DAHKTC010000165.1 | 1424 | 2581 | + |
| *Escherichia coli* | GCA_023783615.1 | DAHKXS010000080.1 | 1403 | 2560 | + |
| *Escherichia coli* | GCA_023783615.1 | DAHKXS010000131.1 | 1444 | 2601 | + |
| *Escherichia coli* | GCA_024611375.1 | DAILGF010000075.1 | 1402 | 2559 | + |
| *Escherichia coli* | GCA_024611375.1 | DAILGF010000127.1 | 1443 | 2600 | + |
| *Escherichia coli* | GCA_024612605.1 | DAILHC010000077.1 | 1381 | 2538 | + |
| *Escherichia coli* | GCA_024612605.1 | DAILHC010000155.1 | 1422 | 2579 | + |
| *Escherichia coli* | GCA_024612725.1 | DAILHH010000120.1 | 1402 | 2559 | + |
| *Escherichia coli* | GCA_024612725.1 | DAILHH010000261.1 | 1443 | 2600 | + |
| *Escherichia coli* | GCA_024613245.1 | DAILIE010000146.1 | 1097 | 2254 | + |
| *Escherichia coli* | GCA_024613245.1 | DAILIE010000299.1 | 1137 | 2294 | + |
| *Escherichia coli* | GCA_024613445.1 | DAILIK010000137.1 | 1252 | 2409 | + |
| *Escherichia coli* | GCA_024613745.1 | DAILJA010000093.1 | 1402 | 2559 | + |
| *Escherichia coli* | GCA_024613745.1 | DAILJA010000140.1 | 1443 | 2600 | + |
| *Escherichia coli* | GCA_024614025.1 | DAILJL010000139.1 | 1213 | 2370 | + |
| *Escherichia coli* | GCA_024614025.1 | DAILJL010000275.1 | 1138 | 2295 | + |
| *Escherichia coli* | GCA_024614045.1 | DAILJO010000062.1 | 1402 | 2559 | + |
| *Escherichia coli* | GCA_024614045.1 | DAILJO010000115.1 | 1443 | 2600 | + |

| | | | | | |
|---|---|---|---|---|---|
| *Escherichia coli* | GCA_024614465.1 | DAILKD010000034.1 | 1129 | 2286 | + |
| *Escherichia coli* | GCA_024614465.1 | DAILKD010000074.1 | 1169 | 2326 | + |
| *Escherichia coli* | GCA_024614745.1 | DAILKQ010000093.1 | 1402 | 2559 | + |
| *Escherichia coli* | GCA_024614745.1 | DAILKQ010000149.1 | 1443 | 2600 | + |
| *Escherichia coli* | GCA_024674005.1 | DAIUSA010000034.1 | 1129 | 2286 | + |
| *Escherichia coli* | GCA_024674005.1 | DAIUSA010000075.1 | 1169 | 2326 | + |
| *Escherichia coli* | GCA_024674155.1 | DAIUSF010000094.1 | 1403 | 2560 | + |
| *Escherichia coli* | GCA_024674155.1 | DAIUSF010000148.1 | 1444 | 2601 | + |
| *Escherichia coli* | GCA_024674175.1 | DAIUSI010000118.1 | 1403 | 2560 | + |
| *Escherichia coli* | GCA_024674175.1 | DAIUSI010000163.1 | 1444 | 2601 | + |
| *Escherichia coli* | GCA_024674585.1 | DAIUTA010000379.1 | 1443 | 2600 | + |
| *Escherichia coli* | GCA_024674565.1 | DAIUTC010000108.1 | 1402 | 2559 | + |
| *Escherichia coli* | GCA_024674565.1 | DAIUTC010000171.1 | 1443 | 2600 | + |
| *Escherichia coli* | GCA_024674645.1 | DAIUTE010000088.1 | 1402 | 2559 | + |
| *Escherichia coli* | GCA_024674645.1 | DAIUTE010000149.1 | 1443 | 2600 | + |
| *Escherichia coli* | GCA_024674665.1 | DAIUTG010000115.1 | 1402 | 2559 | + |
| *Escherichia coli* | GCA_024674665.1 | DAIUTG010000170.1 | 1443 | 2600 | + |
| *Escherichia coli* | GCA_024674955.1 | DAIUTT010000088.1 | 1403 | 2560 | + |
| *Escherichia coli* | GCA_024674955.1 | DAIUTT010000151.1 | 1444 | 2601 | + |
| *Escherichia coli* | GCA_024675295.1 | DAIUUK010000079.1 | 1435 | 2592 | + |
| *Escherichia coli* | GCA_024675295.1 | DAIUUK010000142.1 | 1476 | 2633 | + |
| *Escherichia coli* | GCA_024676435.1 | DAIUWL010000121.1 | 1403 | 2560 | + |
| *Escherichia coli* | GCA_024676435.1 | DAIUWL010000197.1 | 1444 | 2601 | + |
| *Escherichia coli* | GCA_024676515.1 | DAIUWO010000111.1 | 1403 | 2560 | + |
| *Escherichia coli* | GCA_024676515.1 | DAIUWO010000208.1 | 1444 | 2601 | + |
| *Escherichia coli* | GCA_024676535.1 | DAIUWR010000060.1 | 1401 | 2558 | + |
| *Escherichia coli* | GCA_024676535.1 | DAIUWR010000112.1 | 1442 | 2599 | + |
| *Escherichia coli* | GCA_024677385.1 | DAIUXD010000085.1 | 1383 | 2540 | + |
| *Escherichia coli* | GCA_024677385.1 | DAIUXD010000128.1 | 1424 | 2581 | + |
| *Escherichia coli* | GCA_024679815.1 | DAIUXX010000109.1 | 1436 | 2593 | + |
| *Escherichia coli* | GCA_024679815.1 | DAIUXX010000146.1 | 1477 | 2634 | + |
| *Escherichia coli* | GCA_024679845.1 | DAIUXZ010000069.1 | 1402 | 2559 | + |
| *Escherichia coli* | GCA_024679845.1 | DAIUXZ010000105.1 | 1443 | 2600 | + |
| *Escherichia coli* | GCA_024682715.1 | DAIUZT010000037.1 | 1129 | 2286 | + |
| *Escherichia coli* | GCA_024682715.1 | DAIUZT010000107.1 | 1169 | 2326 | + |
| *Escherichia coli* | GCA_024684575.1 | DAIVBA010000093.1 | 1435 | 2592 | + |
| *Escherichia coli* | GCA_024684575.1 | DAIVBA010000142.1 | 1476 | 2633 | + |
| *Escherichia coli* | GCA_024686515.1 | DAIVCK010000110.1 | 1436 | 2593 | + |
| *Escherichia coli* | GCA_024686515.1 | DAIVCK010000147.1 | 1477 | 2634 | + |
| *Escherichia coli* | GCA_024686735.1 | DAIVCY010000038.1 | 3574 | 4731 | + |
| *Escherichia coli* | GCA_024686735.1 | DAIVCY010000087.1 | 1116 | 2273 | + |
| *Escherichia coli* | GCA_024687425.1 | DAIVDP010000090.1 | 1403 | 2560 | + |
| *Escherichia coli* | GCA_024687425.1 | DAIVDP010000146.1 | 1444 | 2601 | + |
| *Escherichia coli* | GCA_024687365.1 | DAIVDS010000089.1 | 1403 | 2560 | + |
| *Escherichia coli* | GCA_024687365.1 | DAIVDS010000144.1 | 1444 | 2601 | + |
| *Klebsiella aerogenes* | GCA_025094595.1 | DAJDBL010000038.1 | 1383 | 2540 | + |
| *Klebsiella aerogenes* | GCA_025094595.1 | DAJDBL010000074.1 | 1424 | 2581 | + |
| *Klebsiella pneumoniae* | GCA_025095235.1 | DAJDCD010000068.1 | 1382 | 2539 | + |
| *Klebsiella pneumoniae* | GCA_025095235.1 | DAJDCD010000131.1 | 1423 | 2580 | + |
| *Klebsiella pneumoniae* | GCA_025102265.1 | DAJDGO010000057.1 | 1383 | 2540 | + |
| *Klebsiella pneumoniae* | GCA_025102265.1 | DAJDGO010000093.1 | 1424 | 2581 | + |
| *Escherichia coli* | GCA_025328385.1 | DAJIBJ010000093.1 | 1117 | 2274 | + |
| *Escherichia coli* | GCA_025328385.1 | DAJIBJ010000144.1 | 1144 | 2301 | + |
| *Escherichia coli* | GCA_025671685.1 | DAJNZE010000112.1 | 937 | 2094 | + |

| | | | | | |
|---|---|---|---|---|---|
| *Escherichia coli* | GCA_025671685.1 | DAJNZE010000182.1 | 964 | 2121 | + |
| *Escherichia coli* | GCA_025671745.1 | DAJNZF010000091.1 | 1372 | 2529 | + |
| *Escherichia coli* | GCA_025671745.1 | DAJNZF010000135.1 | 277 | 1434 | + |
| *Escherichia coli* | GCA_025672085.1 | DAJNZW010000090.1 | 904 | 2061 | + |
| *Escherichia coli* | GCA_025672085.1 | DAJNZW010000167.1 | 1423 | 2580 | + |
| *Escherichia coli* | GCA_025672125.1 | DAJNZX010000107.1 | 1065 | 2222 | + |
| *Escherichia coli* | GCA_025672125.1 | DAJNZX010000180.1 | 992 | 2149 | + |
| *Escherichia coli* | GCA_025672165.1 | DAJOAA010000057.1 | 1058 | 2215 | + |
| *Escherichia coli* | GCA_025672165.1 | DAJOAA010000111.1 | 1089 | 2246 | + |
| *Escherichia coli* | GCA_025672205.1 | DAJOAC010000090.1 | 1382 | 2539 | + |
| *Escherichia coli* | GCA_025672205.1 | DAJOAC010000123.1 | 1423 | 2580 | + |
| *Escherichia coli* | GCA_025672325.1 | DAJOAJ010000093.1 | 979 | 2136 | + |
| *Escherichia coli* | GCA_025672325.1 | DAJOAJ010000179.1 | 1007 | 2164 | + |
| *Escherichia coli* | GCA_025672505.1 | DAJOAR010000140.1 | 1020 | 2177 | + |
| *Escherichia coli* | GCA_025672505.1 | DAJOAR010000205.1 | 1118 | 2275 | + |
| *Escherichia coli* | GCA_025674805.1 | DAJOBW010000081.1 | 909 | 2066 | + |
| *Escherichia coli* | GCA_025674805.1 | DAJOBW010000147.1 | 1089 | 2246 | + |
| *Escherichia coli* | GCA_025675265.1 | DAJOCG010000065.1 | 1382 | 2539 | + |
| *Escherichia coli* | GCA_025675265.1 | DAJOCG010000118.1 | 1423 | 2580 | + |
| *Escherichia coli* | GCA_025675245.1 | DAJOCJ010000073.1 | 1114 | 2271 | + |
| *Escherichia coli* | GCA_025675245.1 | DAJOCJ010000144.1 | 1145 | 2302 | + |
| *Escherichia coli* | GCA_025877765.1 | DAJQNA010000175.1 | 1002 | 2159 | + |
| *Escherichia coli* | GCA_025877765.1 | DAJQNA010000254.1 | 1003 | 2160 | + |
| *Escherichia coli* | GCA_026314675.1 | DAJZWD010000090.1 | 1381 | 2538 | + |
| *Escherichia coli* | GCA_026314675.1 | DAJZWD010000170.1 | 1422 | 2579 | + |
| *Escherichia coli* | GCA_026348895.1 | DAKBWI010000090.1 | 1381 | 2538 | + |
| *Escherichia coli* | GCA_026348895.1 | DAKBWI010000179.1 | 1348 | 2505 | + |
| *Escherichia coli* | GCA_026348915.1 | DAKBWK010000070.1 | 1383 | 2540 | + |
| *Escherichia coli* | GCA_026348915.1 | DAKBWK010000120.1 | 1424 | 2581 | + |
| *Escherichia coli* | GCA_026348995.1 | DAKBWM010000082.1 | 973 | 2130 | + |
| *Escherichia coli* | GCA_026348995.1 | DAKBWM010000136.1 | 1012 | 2169 | + |
| *Escherichia coli* | GCA_027361615.1 | DALSPI010000072.1 | 1403 | 2560 | + |
| *Escherichia coli* | GCA_027361615.1 | DALSPI010000137.1 | 21 | 1178 | + |
| *Escherichia coli* | GCA_028829915.1 | DANMJS010000045.1 | 1491 | 2648 | + |
| *Escherichia coli* | GCA_028829915.1 | DANMJS010000096.1 | 1170 | 2327 | + |
| *Escherichia coli* | GCA_028830155.1 | DANMJT010000094.1 | 1098 | 2255 | + |
| *Escherichia coli* | GCA_028830155.1 | DANMJT010000159.1 | 1136 | 2293 | + |
| *Escherichia coli* | GCA_028830425.1 | DANMKC010000064.1 | 1279 | 2436 | + |
| *Escherichia coli* | GCA_028830425.1 | DANMKC010000144.1 | 1166 | 2323 | + |
| *Escherichia coli* | GCA_028830365.1 | DANMKD010000122.1 | 1403 | 2560 | + |
| *Escherichia coli* | GCA_028830365.1 | DANMKD010000178.1 | 1444 | 2601 | + |
| *Escherichia coli* | GCA_028830215.1 | DANMKE010000068.1 | 1098 | 2255 | + |
| *Escherichia coli* | GCA_028830215.1 | DANMKE010000128.1 | 1138 | 2295 | + |
| *Escherichia coli* | GCA_028830235.1 | DANMKF010000120.1 | 1403 | 2560 | + |
| *Escherichia coli* | GCA_028830235.1 | DANMKF010000179.1 | 1444 | 2601 | + |
| *Escherichia coli* | GCA_028830235.1 | DANMKF010000180.1 | 1444 | 2601 | + |
| *Escherichia coli* | GCA_028830865.1 | DANMKK010000090.1 | 1381 | 2538 | + |
| *Escherichia coli* | GCA_028830865.1 | DANMKK010000137.1 | 1422 | 2579 | + |
| *Escherichia coli* | GCA_028922145.1 | DANNVK010000096.1 | 1098 | 2255 | + |
| *Escherichia coli* | GCA_028922145.1 | DANNVK010000144.1 | 1138 | 2295 | + |
| *Escherichia coli* | GCA_029469365.1 | DAODFY010000097.1 | 1097 | 2254 | + |
| *Escherichia coli* | GCA_029469365.1 | DAODFY010000180.1 | 1135 | 2292 | + |
| *Escherichia coli* | GCA_029516395.1 | DAOETM010000081.1 | 1008 | 2165 | + |
| *Escherichia coli* | GCA_029516395.1 | DAOETM010000145.1 | 1029 | 2186 | + |

| | | | | | |
|---|---|---|---|---|---|
| *Escherichia coli* | GCA_029706505.1 | DAOJAP010000142.1 | 1336 | 2493 | + |
| *Escherichia coli* | GCA_029706505.1 | DAOJAP010000193.1 | 1169 | 2326 | + |
| *Escherichia coli* | GCA_031778115.1 | DAPNUU010000168.1 | 1403 | 2560 | + |
| *Escherichia coli* | GCA_031778115.1 | DAPNUU010000235.1 | 1177 | 2334 | + |
| *Escherichia coli* | GCA_032285135.1 | DAPXYT010000087.1 | 1128 | 2285 | + |
| *Escherichia coli* | GCA_032285135.1 | DAPXYT010000166.1 | 402 | 1559 | + |
| *Escherichia coli* | GCA_032285135.1 | DAPXYT010000167.1 | 402 | 1559 | + |
| *Escherichia coli* | GCA_032285205.1 | DAPXYU010000077.1 | 1490 | 2647 | + |
| *Escherichia coli* | GCA_032285205.1 | DAPXYU010000130.1 | 1169 | 2326 | + |
| *Escherichia coli* | GCA_032285685.1 | DAPXZH010000037.1 | 1129 | 2286 | + |
| *Escherichia coli* | GCA_032285685.1 | DAPXZH010000079.1 | 1169 | 2326 | + |
| *Escherichia coli* | GCA_032285685.1 | DAPXZH010000080.1 | 1169 | 2326 | + |
| *Escherichia coli* | GCA_032285765.1 | DAPXZI010000061.1 | 1491 | 2648 | + |
| *Escherichia coli* | GCA_032285765.1 | DAPXZI010000107.1 | 1169 | 2326 | + |
| *Escherichia coli* | GCA_032285705.1 | DAPXZJ010000156.1 | 1096 | 2253 | + |
| *Escherichia coli* | GCA_032285705.1 | DAPXZJ010000262.1 | 1137 | 2294 | + |
| *Escherichia coli* | GCA_032285745.1 | DAPXZK010000119.1 | 1128 | 2285 | + |
| *Escherichia coli* | GCA_032285745.1 | DAPXZK010000205.1 | 1169 | 2326 | + |
| *Escherichia coli* | GCA_032286305.1 | DAPYAA010000118.1 | 1127 | 2284 | + |
| *Escherichia coli* | GCA_032286305.1 | DAPYAA010000201.1 | 1168 | 2325 | + |
| *Escherichia coli* | GCA_032286945.1 | DAPYBC010000099.1 | 1138 | 2295 | + |
| *Escherichia coli* | GCA_032286945.1 | DAPYBC010000170.1 | 1137 | 2294 | + |
| *Escherichia coli* | GCA_032310475.1 | DAPZBO010000135.1 | 892 | 2049 | + |
| *Escherichia coli* | GCA_032310475.1 | DAPZBO010000270.1 | 942 | 2099 | + |
| *Escherichia coli* | GCA_032572315.1 | DAQDDW010000064.1 | 1403 | 2560 | + |
| *Escherichia coli* | GCA_032572315.1 | DAQDDW010000121.1 | 1444 | 2601 | + |
| *Escherichia coli* | GCA_032633685.1 | DAQDXW010000042.1 | 1547 | 2704 | + |
| *Escherichia coli* | GCA_032633685.1 | DAQDXW010000090.1 | 1423 | 2580 | + |
| *Escherichia coli* | GCA_032633665.1 | DAQDXX010000035.1 | 1383 | 2540 | + |
| *Escherichia coli* | GCA_032633665.1 | DAQDXX010000066.1 | 1424 | 2581 | + |
| *Escherichia coli* | GCA_033023035.1 | DAQMUO010000076.1 | 1383 | 2540 | + |
| *Escherichia coli* | GCA_033023035.1 | DAQMUO010000127.1 | 1424 | 2581 | + |
| *Escherichia coli* | GCA_033406835.1 | DARBPF010000057.1 | 1714 | 2871 | + |
| *Escherichia coli* | GCA_033406835.1 | DARBPF010000151.1 | 1423 | 2580 | + |
| *Escherichia coli* | GCA_033407035.1 | DARBPJ010000092.1 | 1383 | 2540 | + |
| *Escherichia coli* | GCA_033407035.1 | DARBPJ010000161.1 | 1424 | 2581 | + |
| *Escherichia coli* | GCA_033407275.1 | DARBPY010000031.1 | 1714 | 2871 | + |
| *Escherichia coli* | GCA_033407275.1 | DARBPY010000066.1 | 1755 | 2912 | + |
| *Escherichia coli* | GCA_019371395.1 | JAEMZP010000064.1 | 1462 | 2616 | + |
| *Escherichia coli* | GCA_019370195.1 | JAEOBM010000043.1 | 1715 | 2869 | + |
| *Escherichia coli* | GCA_019366595.1 | JAEOHT010000098.1 | 1461 | 2615 | + |
| *Escherichia coli* | GCA_019366165.1 | JAEOIR010000073.1 | 4886 | 6040 | - |
| *Escherichia coli* | GCA_019365285.1 | JAEOKV010000074.1 | 7778 | 8932 | + |
| *Escherichia coli* | GCA_028755825.1 | JAIQMT010000115.1 | 1123 | 2277 | - |
| *Escherichia coli* | GCA_028756175.1 | JAIQNL010000152.1 | 1468 | 2622 | + |
| *Escherichia coli* | GCA_023095725.1 | JALLYW010000032.1 | 1207 | 2361 | + |
| *Escherichia coli* | GCA_023095905.1 | JALLZG010000090.1 | 1207 | 2361 | + |
| *Escherichia coli* | GCA_023095915.1 | JALLZH010000044.1 | 2374 | 3528 | + |
| *Escherichia coli* | GCA_023095935.1 | JALLZJ010000090.1 | 1207 | 2361 | + |
| *Escherichia coli* | GCA_023097055.1 | JALMAQ010000071.1 | 1172 | 2326 | + |
| *Escherichia coli* | GCA_023097235.1 | JALMBB010000114.1 | 301 | 1455 | - |
| *Salmonella enterica* | GCA_030094115.1 | JAMUIC010000030.1 | 224 | 1378 | - |
| *Escherichia coli* | GCA_024754685.1 | JANTIV010000066.1 | 1310 | 2464 | - |
| *Escherichia coli* | GCA_024754805.1 | JANTKT010000093.1 | 332 | 1486 | - |

| | | | | | |
|---|---|---|---|---|---|
| *Escherichia coli* | GCA_024755625.1 | JANTLF010000083.1 | 1144 | 2298 | + |
| *Escherichia coli* | GCA_030712625.1 | JAUOXB010000107.1 | 301 | 1455 | - |
| *Escherichia coli* | GCA_030763085.1 | JAUOYY010000053.1 | 1175 | 2329 | + |
| *Escherichia coli* | GCA_032661265.1 | JAVADQ010000071.1 | 1240 | 2394 | + |
| *Escherichia coli* | GCA_032661065.1 | JAVADU010000053.1 | 4619 | 5773 | - |
| *Escherichia coli* | GCA_032661005.1 | JAVADX010000105.1 | 335 | 1489 | - |
| *Escherichia coli* | GCA_032660985.1 | JAVADZ010000050.1 | 8419 | 9573 | + |

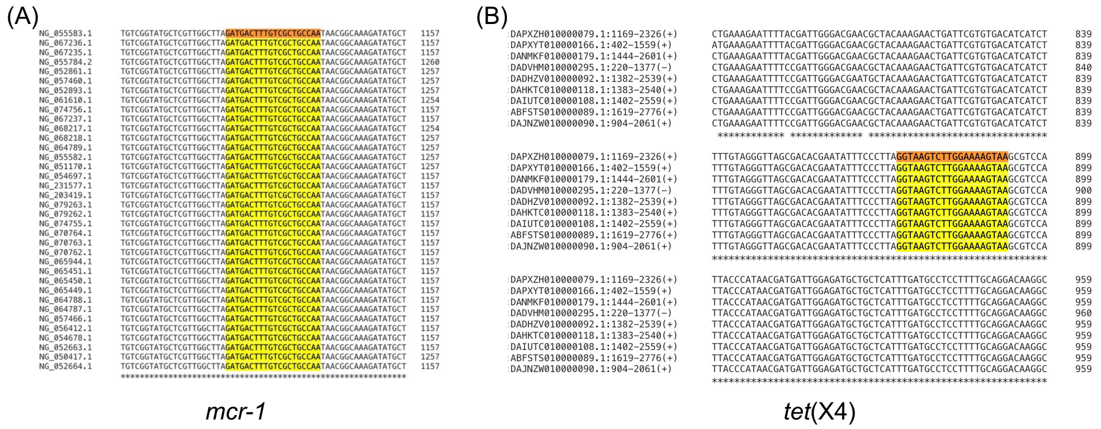


Fig. S1 Conservation analysis of the *mcr-1* and *tet*(X4) protospacer sequences. The 36 known *mcr-1* variants were analyzed (A). The 383 available *tet*(X4) sequences were downloaded from MicroBIGG-E database, and 9 variants were identified using cd-hit-est analysis (B). The protospacers (highlighted regions) were conserved among the sequences.

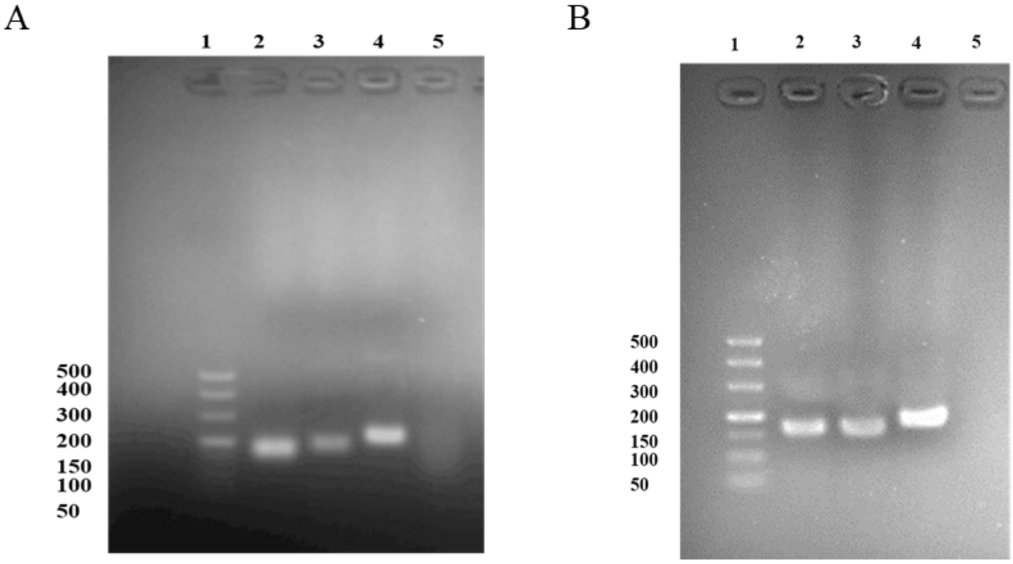


Fig. S2 Electrophoresis results of the *mcr-1* (A) and *tet*(X4) (B) RPA amplification products, respectively.

Note: 1: Marker 500; 2: The amplification products with the pair set 1; 3: The amplification products with the pair set 2; 4: The amplification products with the pair set 3; 5: Negative control.

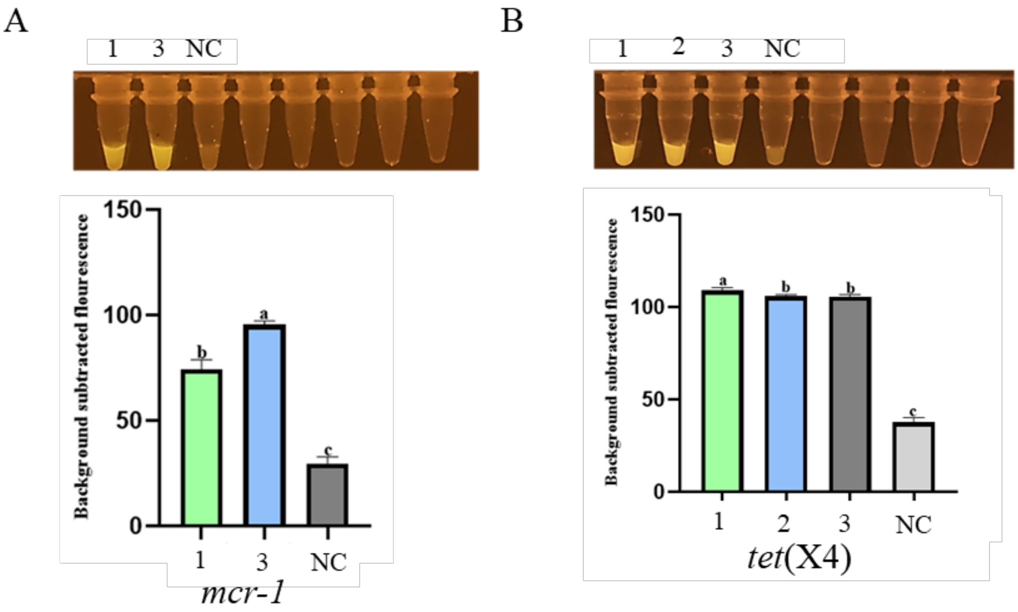


Fig. S3 Efficiency and fluorescence intensity of different RPA primer sets to activate the CRISPR-Cas12b detection systems. Different letters on the top of columns denote significant differences ($P < 0.05$, ANOVA). Error bars represent means ± SEM (n = 3 replicates).

Note: 1: The pair set 1; 2: The pair set 2; 3: The pair set 3; NC: Negative control.

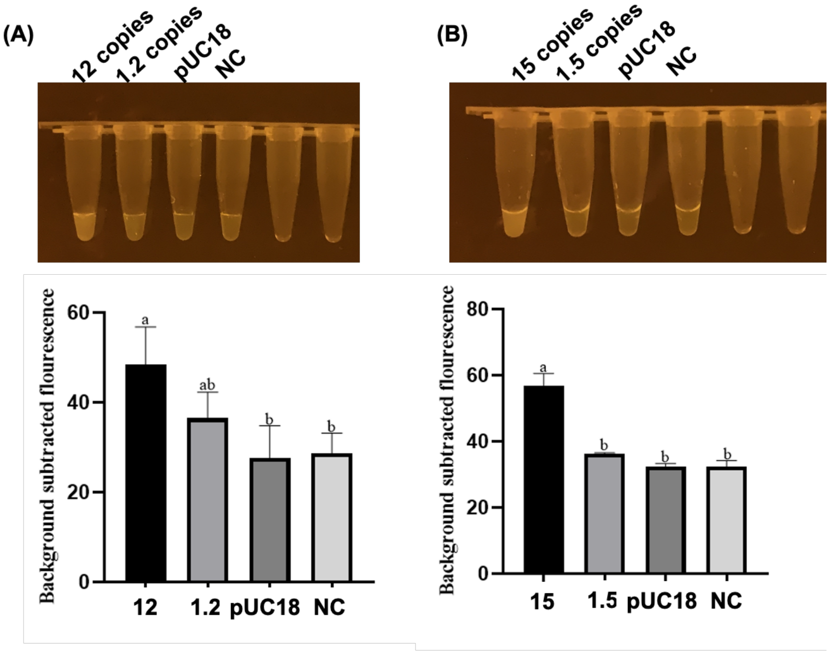


Fig. S4 the sensitivity assessment and fluorescence intensity of the one-tube RPA-CRISPR-Cas12b-based *mcr-1* (A) and *tet*(X4) (B) detection system. pUC18 plasmid (the vector used for *mcr-1* and *tet*(X4) dilution) was included in the assay. NC: negative control. Different letters on the top of columns denote significant differences ($P < 0.05$, ANOVA). Error bars represent means ± SEM (n = 3 replicates). The 'Background subtracted fluorescence' values were fluorescence values recorded in the reaction tubes minus the average florescence value obtained

from three blank tubes.

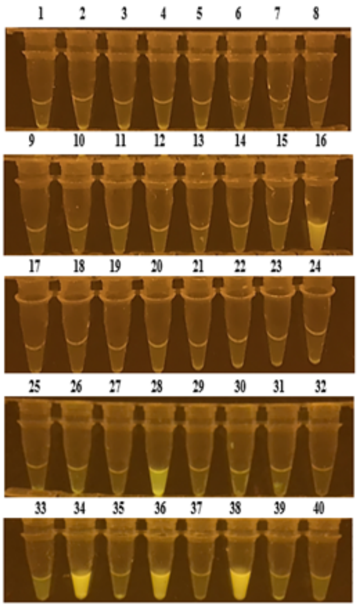


| | 1 | 2 | 3 | 4 | 5 |
|---|---|---|---|---|---|
| CRISPR | - | - | - | - | - |
| Culture | - | - | - | - | - |
| | 6 | 7 | 8 | 9 | 10 |
| CRISPR | - | - | - | - | - |
| Culture | - | - | - | - | - |
| | 11 | 12 | 13 | 14 | 15 |
| CRISPR | - | - | - | - | - |
| Culture | - | - | - | - | - |
| | 16 | 17 | 18 | 19 | 20 |
| CRISPR | + | - | - | - | - |
| Culture | - | - | - | - | - |
| | 21 | 22 | 23 | 24 | 25 |
| CRISPR | - | - | - | - | - |
| Culture | - | - | - | - | - |
| | 26 | 27 | 28 | 29 | 30 |
| CRISPR | - | - | + | - | - |
| Culture | - | - | - | - | - |
| | 31 | 32 | 33 | 34 | 35 |
| CRISPR | - | - | + | + | - |
| Culture | - | - | - | + | - |
| | 36 | 37 | 38 | 39 | 40 |
| CRISPR | + | - | + | - | - |
| Culture | + | - | + | - | - |

+:positive ,-:negative

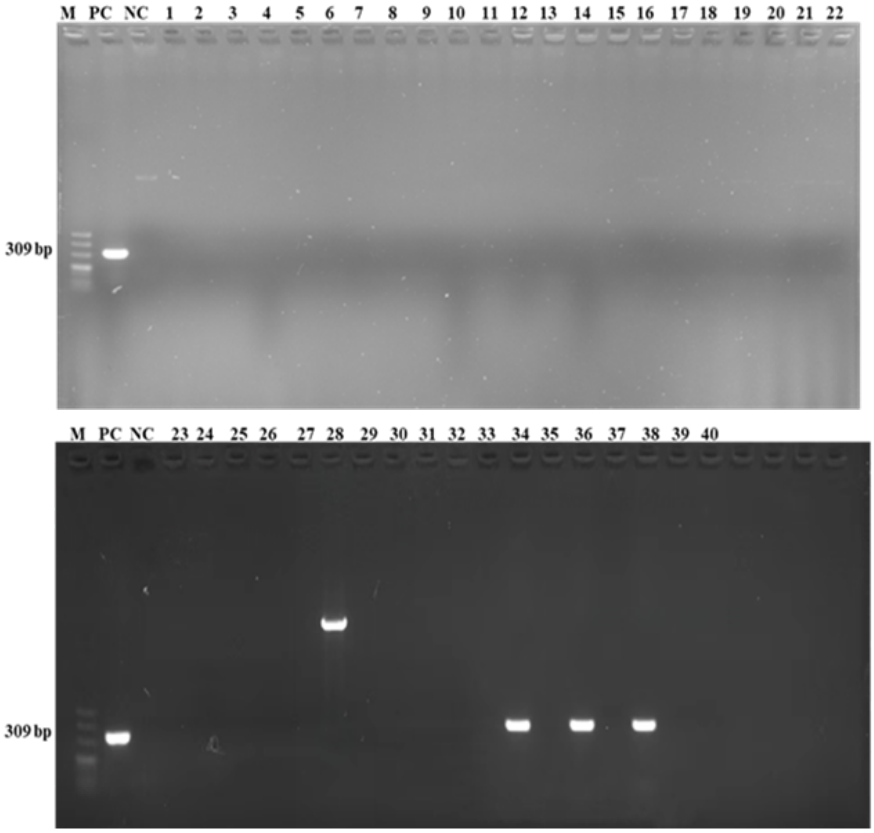


Fig. S5 Detection results of clinical samples by the RPA-CRISPR-Cas12b-based *mcr-1* detection system and the conventional cultivation-based method. Typical single colonies with inconsistent morphology (~20 colonies) were selected by colony PCR to determine the presence of *mcr-1*. The expected size was 309 bp for the *mcr-1* PCR products, and the band in sample 28 was a contamination.

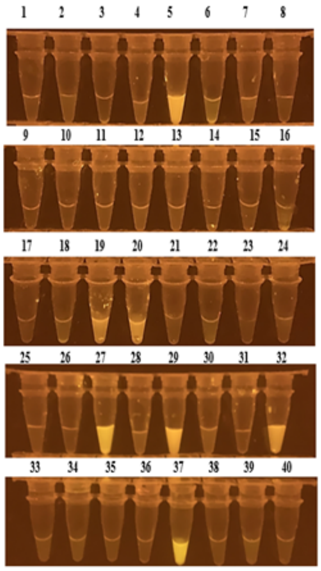


| | | | | | |
|---|---|---|---|---|---|
| | 1 | 2 | 3 | 4 | 5 |
| CRISPR | - | - | - | - | + |
| Culture | - | - | - | - | + |
| | 6 | 7 | 8 | 9 | 10 |
| CRISPR | - | - | - | - | - |
| Culture | - | - | - | - | - |
| | 11 | 12 | 13 | 14 | 15 |
| CRISPR | - | - | - | - | - |
| Culture | - | - | - | - | - |
| | 16 | 17 | 18 | 19 | 20 |
| CRISPR | - | - | - | + | + |
| Culture | - | - | - | + | + |
| | 21 | 22 | 23 | 24 | 25 |
| CRISPR | - | - | - | - | - |
| Culture | - | - | - | - | - |
| | 26 | 27 | 28 | 29 | 30 |
| CRISPR | - | + | - | + | - |
| Culture | - | + | - | + | - |
| | 31 | 32 | 33 | 34 | 35 |
| CRISPR | - | + | - | - | - |
| Culture | - | + | - | - | - |
| | 36 | 37 | 38 | 39 | 40 |
| CRISPR | - | + | - | - | - |
| Culture | - | + | - | - | - |

+:positive ,-:negative

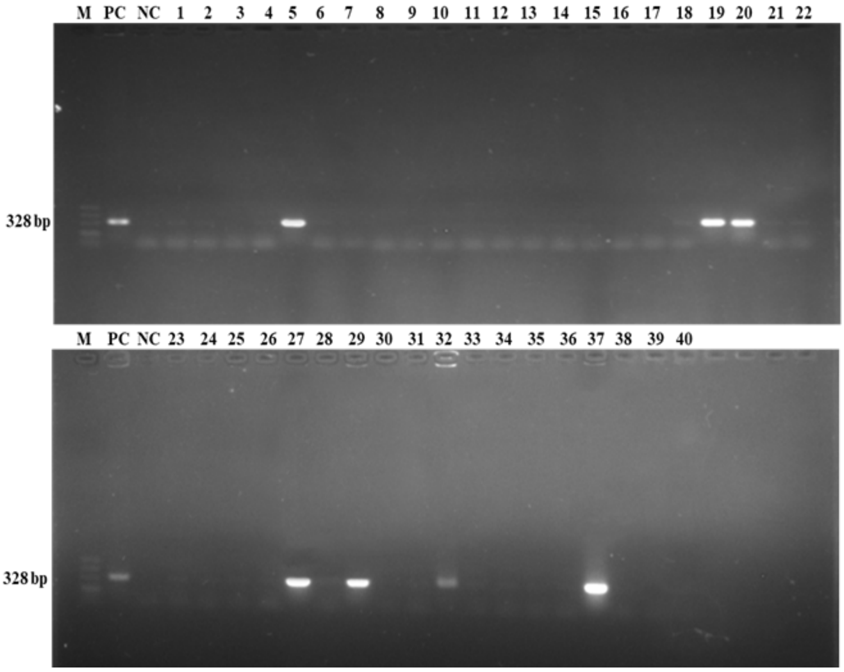


Fig. S6 Detection results of clinical samples by the RPA-CRISPR-Cas12b-based *tet*(X4) detection system and the conventional cultivation-based method. Typical single colonies with inconsistent morphology (~20 colonies) were selected by colony PCR to determine the presence of *tet*(X4).
